# Supplementary material for: Benzoxazole-derivatives enhance progranulin expression and reverse the aberrant lysosomal proteome caused by GRN haploinsufficiency
Source: Nat Commun. 2024 Jul 20;15:6125. doi: 10.1038/s41467-024-50076-8 (PMC11271458; doi:10.1038/s41467-024-50076-8)
Supplement: Supplementary file 1 — Supplementary Information [file 41467_2024_50076_MOESM1_ESM.pdf]

Supplementary Information File

Rachel Tesla[1,2,†], Charlotte Guhl[3,†], Gordon C. Werthmann[1,2,†], Danielle Dixon[1,2], Basar Cenik[1,2], Yesu Addepalli[4], Jue Liang[4], Daniel M. Fass[5], Zachary Rosenthal[6,7], Stephen J. Haggarty[7], Noelle S. Williams[4], Bruce A. Posner[4], Joseph M. Ready[4], Joachim Herz[1,2,8,9]

Affiliations

[1]Department of Molecular Genetics, University of Texas Southwestern Medical Center, Dallas, TX, United States

[2]Center for Translational Neurodegeneration Research, Dallas, TX, United States

[3]Faculty of Chemistry and Earth Sciences, Institute of Organic Chemistry and Macromolecular Chemistry, Friedrich Schiller University Jena, Humboldtstraße 10, 07743 Jena, Germany

[4]Department of Biochemistry, University of Texas Southwestern Medical Center, Dallas, TX, United States

[5]Chemical Neurobiology Laboratory, Center for Genomic Medicine, Departments of Neurology and Psychiatry, Massachusetts General Hospital, Harvard Medical School, Boston, MA, United States

[6]Chemical Neurobiology Laboratory, Center for Genomic Medicine, Massachusetts General Hospital, Boston, United States

[7]Department of Chemistry & Chemical Biology, Harvard University, Cambridge, MA, United States

[8]Department of Neuroscience, University of Texas Southwestern Medical Center, Dallas, TX, United States

[9]Department of Neurology and Neurotherapeutics, University of Texas Southwestern Medical Center, Dallas, TX, United States

[†]These authors contributed equally to this work

Joachim.Herz@utsouthwestern.edu

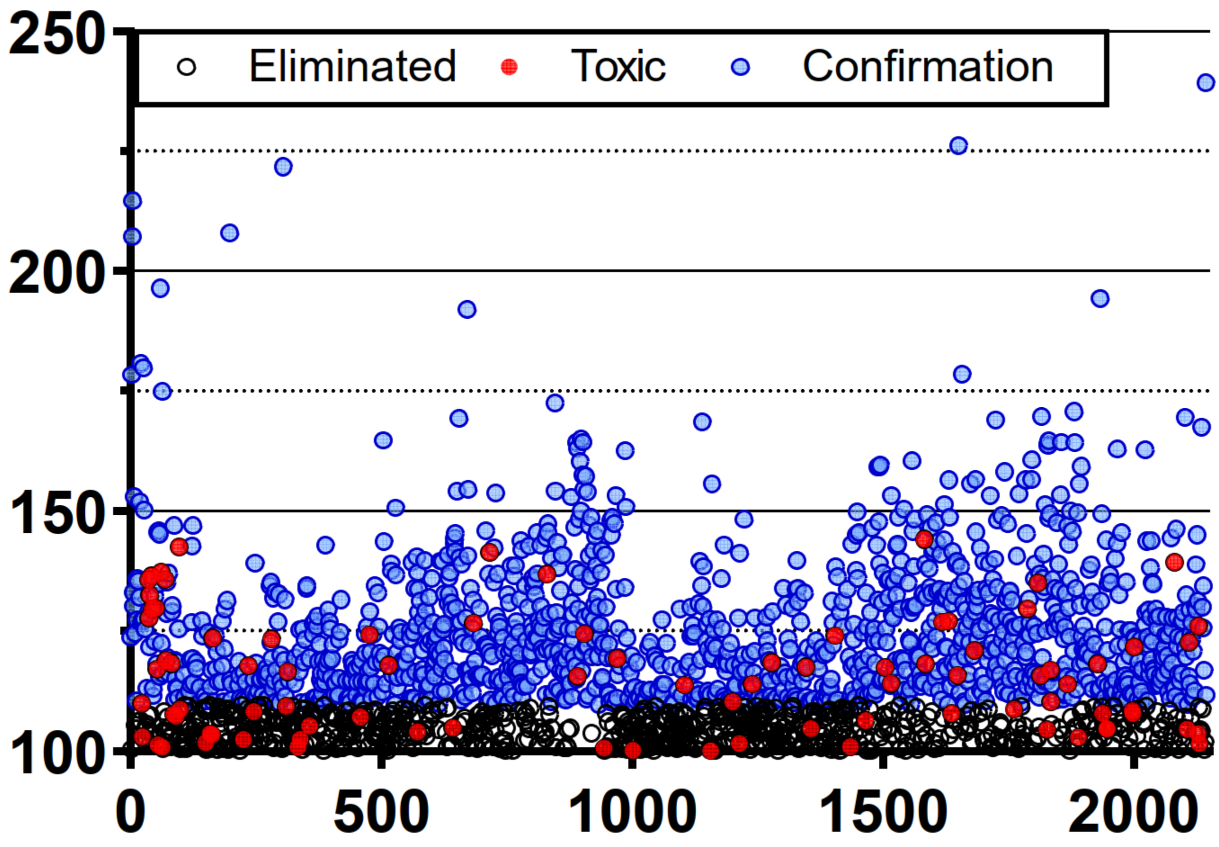

Supplementary Figure 1. Selected molecules from HTS screen and elimination of toxic compounds. 2144 compounds marked in black or red had equal to or greater activity than positive control in HTS. 87 compounds marked in red were eliminated due to suspected toxicity. Dose-response follow-up analysis of top 1280 hits using *GRN* luciferase-based assay (blue).

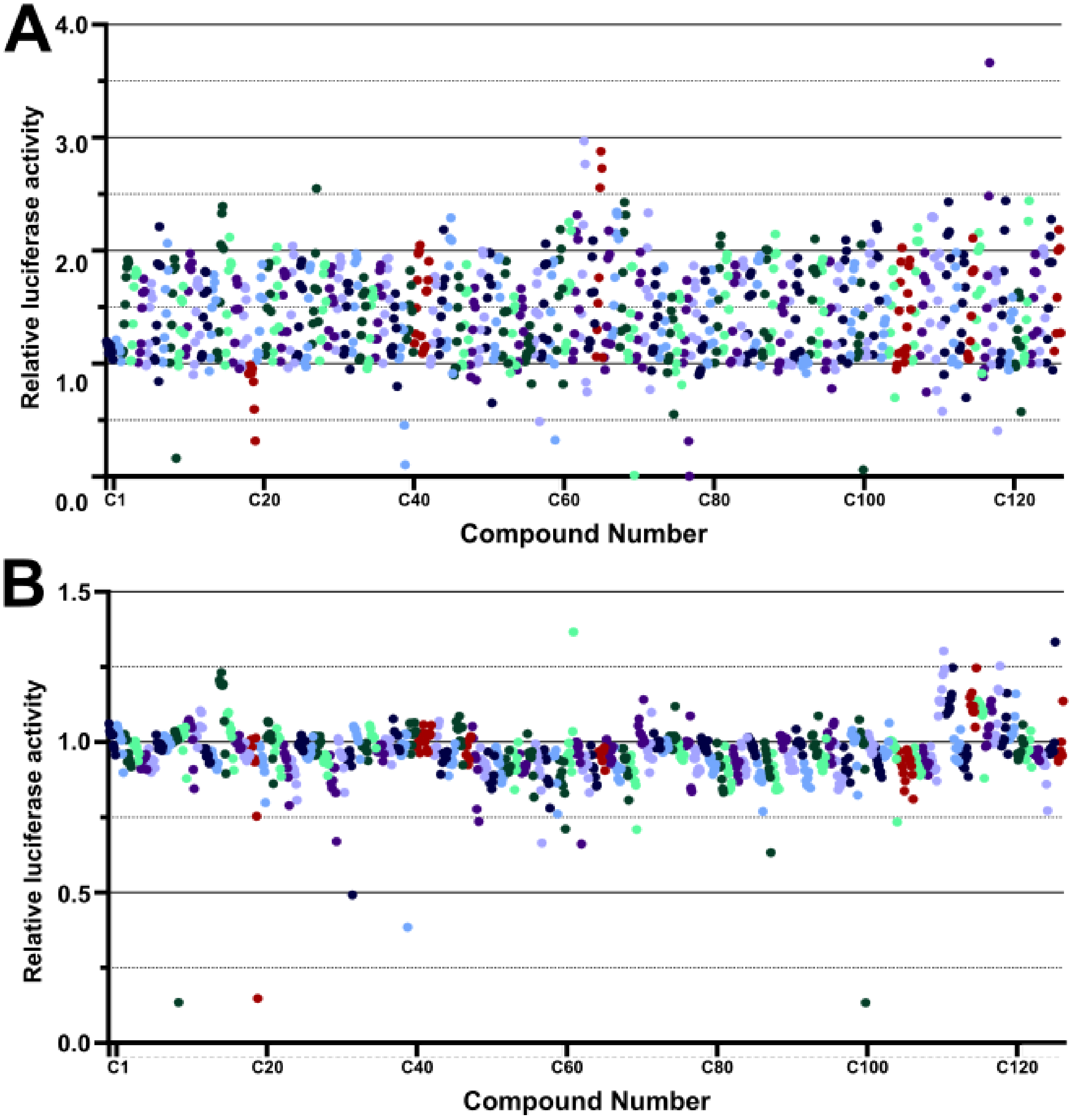

Supplementary Figure 2. In vitro evaluation of 127 compounds in *Grn* luciferase reporter assay and ATP-based toxicity assay. Tested compounds marked in alternating colors, compounds that induced significant increases in PGRN protein marked in red. **(A)** Changes in N2A *Grn* luciferase reporter output for each compound when treated with an eight-concentration dose responses from 10 nM to 30 µM. (n=1) **(B)** Toxicity of each compound over 8 concentrations based on cellular ATP concentration. (n=1)

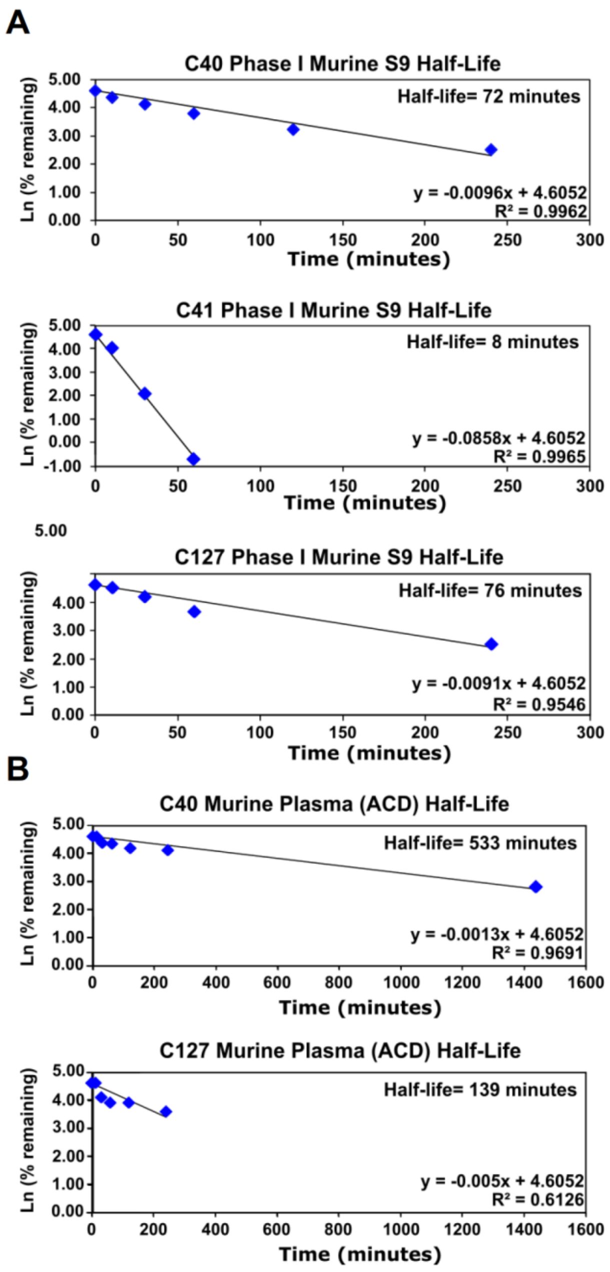

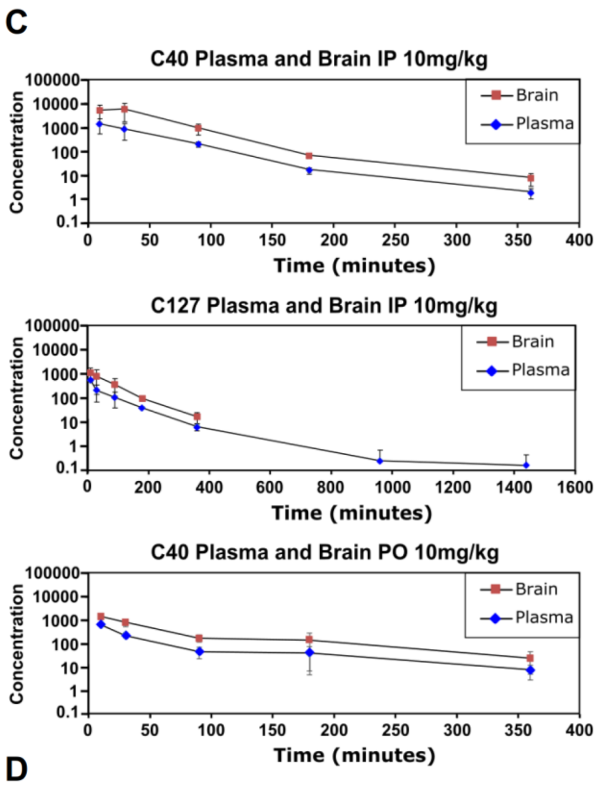

D

IP delivery			
Original		Analog	
C40	10mg/kg	A21	30mg/kg
C_{MAX}	6246.00	C_{MAX}	1480.46
T_{MAX}	30	T_{MAX}	10
$T_{1/2}$	41.28	$T_{1/2}$	119
C127	10mg/kg	A41	25mg/kg
C_{MAX}	1112.00	C_{MAX}	2282.43
T_{MAX}	10	T_{MAX}	10
$T_{1/2}$	58.75	$T_{1/2}$	98

Supplementary Figure 3. In vitro and in vivo PK compound analysis. (A) Liver metabolism assay**.** C40 and C127 had satisfactory half-lives, while C41 had an unsatisfactorily low half-life. (n=1) **(B)** Plasma stability assay. A41 was not evaluated due to high liver metabolism. C40 and C127 had excellent plasma stability, but C40 displayed a significantly higher half-life than C127. (n=1) **(C)** C40 (n=3) and C127 (n=3) exhibited a higher level of drug penetrance into the brain as compared to plasma. C127 displayed a much lower overall drug uptake but persisted in the plasma at low levels for an extended time. PO administration of C40 (n=3) decreased the overall drug uptake, but a similar brain to plasma ratio was observed. **(D)** Original vs. analog in vivo PK

parameters. C_{max} of analogs ranged between the C_{MAX} of the original compounds. T_{MAX} between sets was comparable, while $T_{1/2}$ for the analogs were approximately 2x higher than the original compounds.

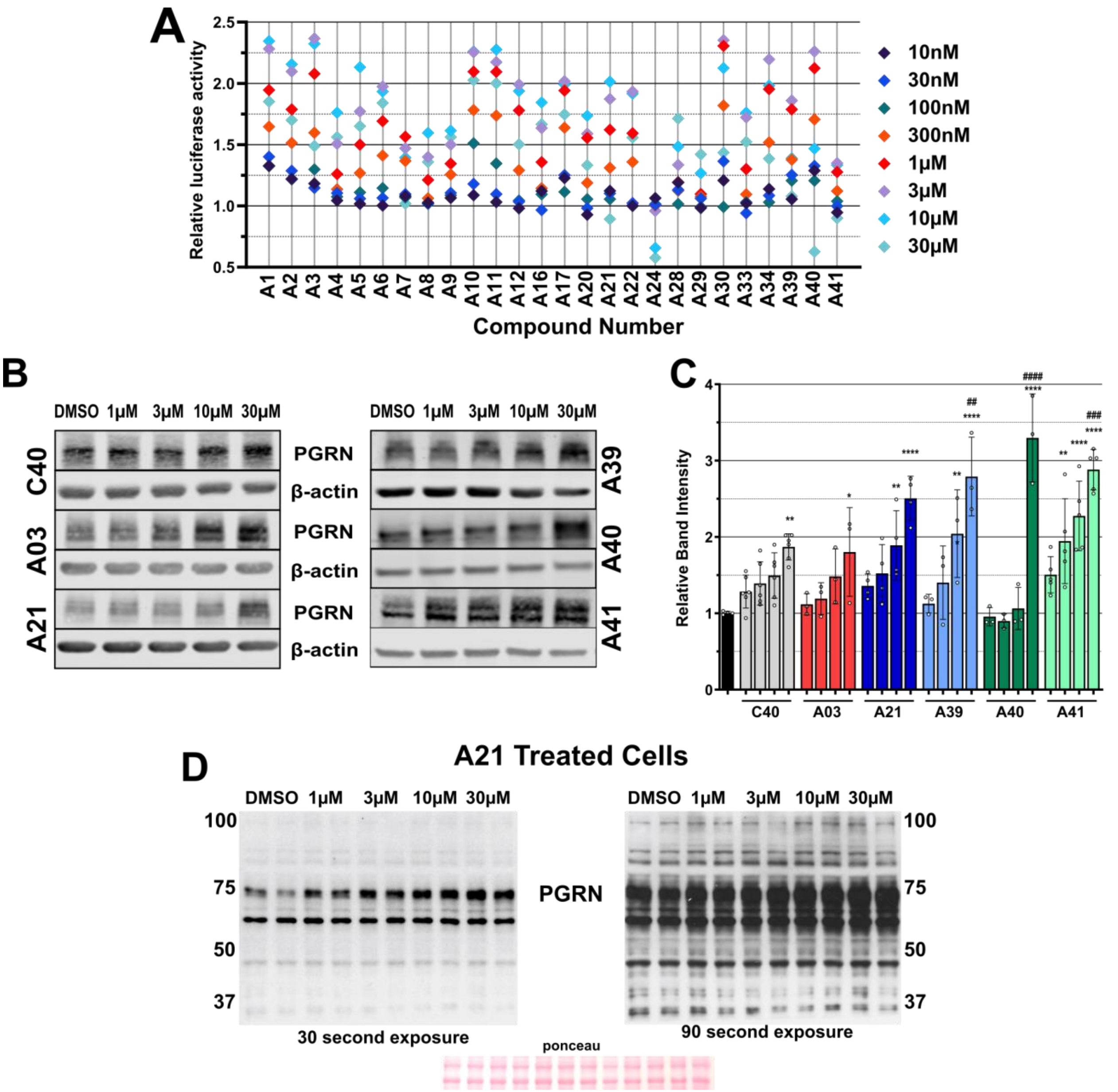

Supplementary Figure 4. Luciferase, immunoblots and quantification of active analogs. **(A)** Compiled data for *Grn* luciferase reporter cells treated with C40 analogs relative to DMSO treated control.(n=1) **(B)** Immunoblot of N2A cells treated with original compound C40, top analog from the first round of synthesis A03, and **subsequently** synthesized analogs. **(C)** Quantification of immunoblots for treated cells. A21 (n=4), A39 (n=3), A40 (n=3), and A41 (n=5) increased PGRN protein significantly more than C40 (n=6) or A03 (n=3) at the highest concentration. Biological replicates are presented as mean values ± SD and an ordinary one-way ANOVA with Dunnett's multiple comparison test was used to test for significance. * represents $p<0.05$, **$p<0.01$, ***$p<0.001$, ****$p<0.0001$ compared to DMSO treatment (n=4); # represents $p<0.05$, $^{\#\#}p<0.01$, $^{\#\#\#}p<0.001$, $^{\#\#\#\#}p<0.0001$ compared to C40 30μM treatment **(D)** Full gel of immunoblot of N2A

cells treated with increasing A21. Two exposures are shown to better visualize lighter bands. Only PGRN bands at 75kDa show a consistent, patterned response to treatment.

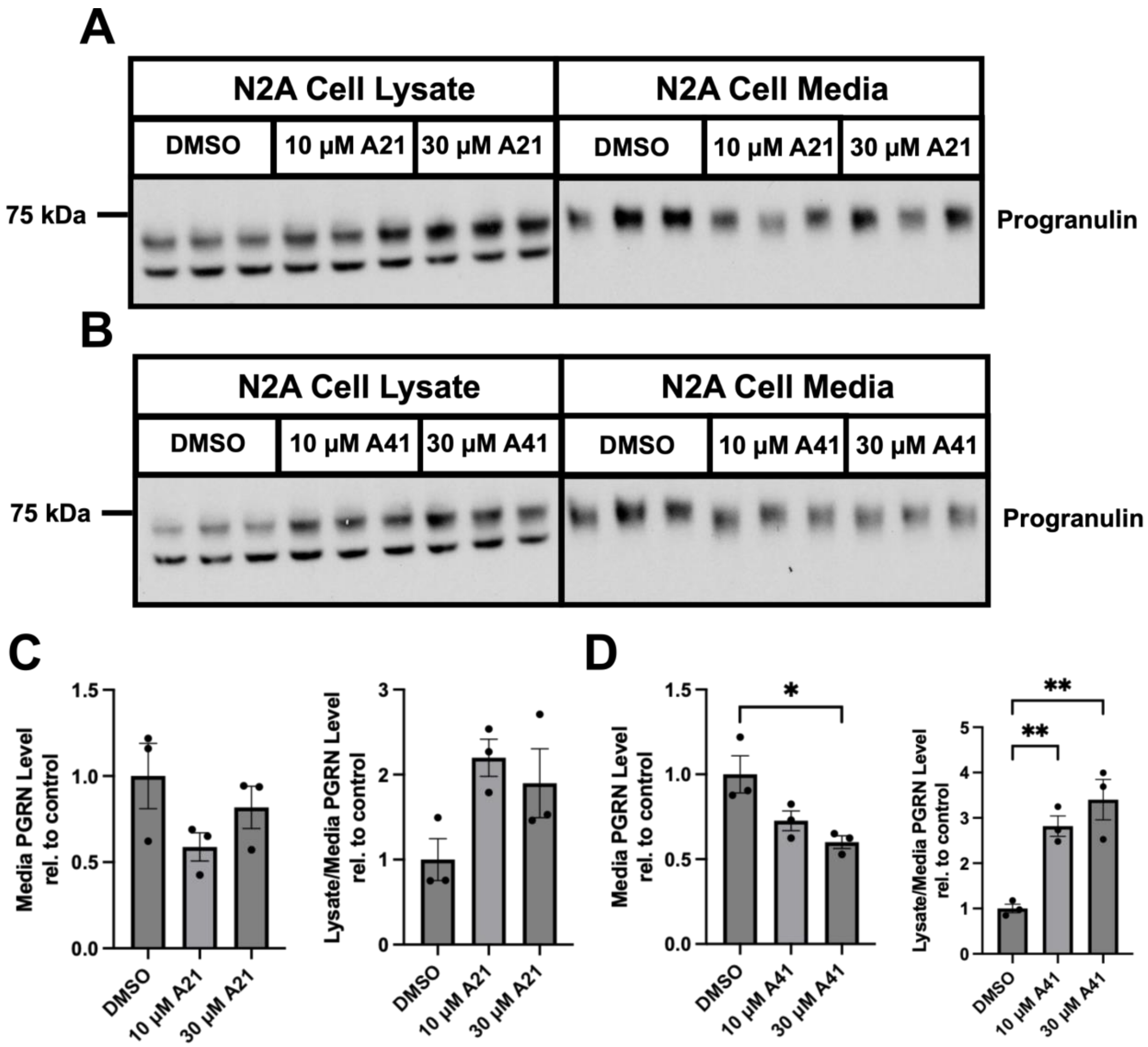

Supplementary Figure 5. Comparison of secreted and intracellular progranulin after analog treatment. (A-B) Progranulin levels in N2A cell lysate and corresponding conditioned media after 24-hour treatment with varying concentrations (0 µM, 10 µM, or 30 µM) of A21 (n=3 each) **(A)** or A41 (n=3 each) **(B)**. **(C-D)** Quantification of progranulin in conditioned media **(C)** and the ratio of progranulin in cell lysate compared to progranulin in conditioned media **(D)**. (ordinary one-way ANOVA with Dunnet's multiple comparison; *p<0.05, **p<0.01, n=3 for all conditions).

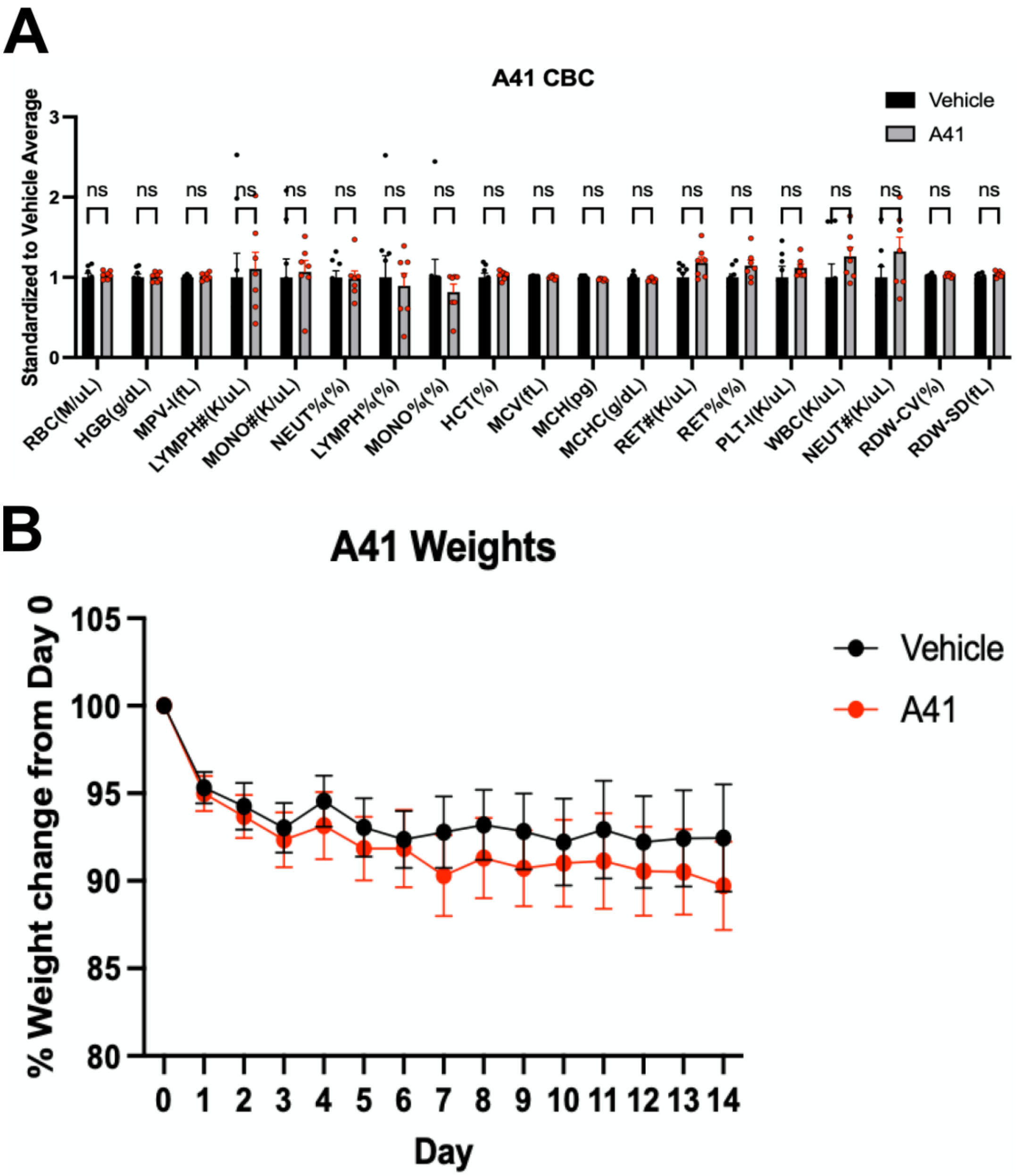

Supplementary Figure 6. Safety analysis of 14-day analog treatment. (A) Complete blood count (CBC) levels of all analyzed parameters are unaffected between mice treated with vehicle (n=8) and A41 (n=7) (multiple t-tests followed by a Two-stage step-up Benjamini, Krieger, and Yekutieli correction, bars represent mean values ± SEM). **(B)** Weights are unaffected between vehicle and A41 treated mice at all timepoints (repeated measures two-way ANOVA, values are represented as mean value ± SEM). RBC = red blood cells, HGB = hemoglobin, MPV = mean platelet volume, LYMPH# = lymphocyte number, MONO# = monocyte number, NEUT% = neutrophil percentage, LYMPH% = lymphocyte percentage, MONO% = monocyte percentage,

HCT = hematocrit, MCV = mean corpuscular volume, MCH = mean corpuscular hemoglobin, MCHC = mean corpuscular hemoglobin concentration, RET# = reticulocyte number, RET% = reticulocyte percent, PLT = platelets, WBC = white blood cells, NEUT# = neutrophil number, RDW = red cell distribution width.

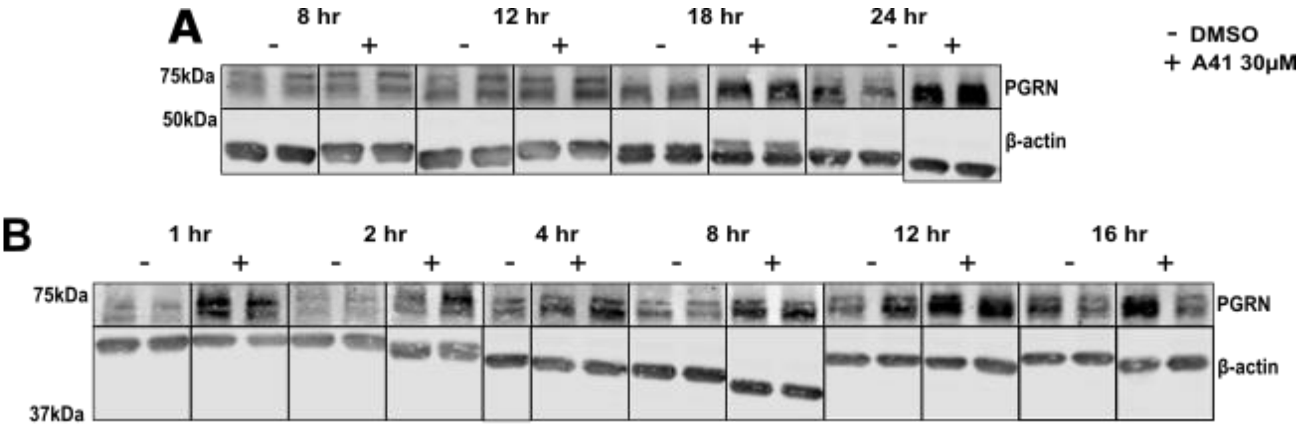

Supplementary Figure 7. Immunoblots of treated N2A cells. (A) Immunoblots of treated N2A cells harvested at increasing time points after treatment. **(B)** Immunoblots of N2A cells harvested at increasing time points after removal of treatment media.

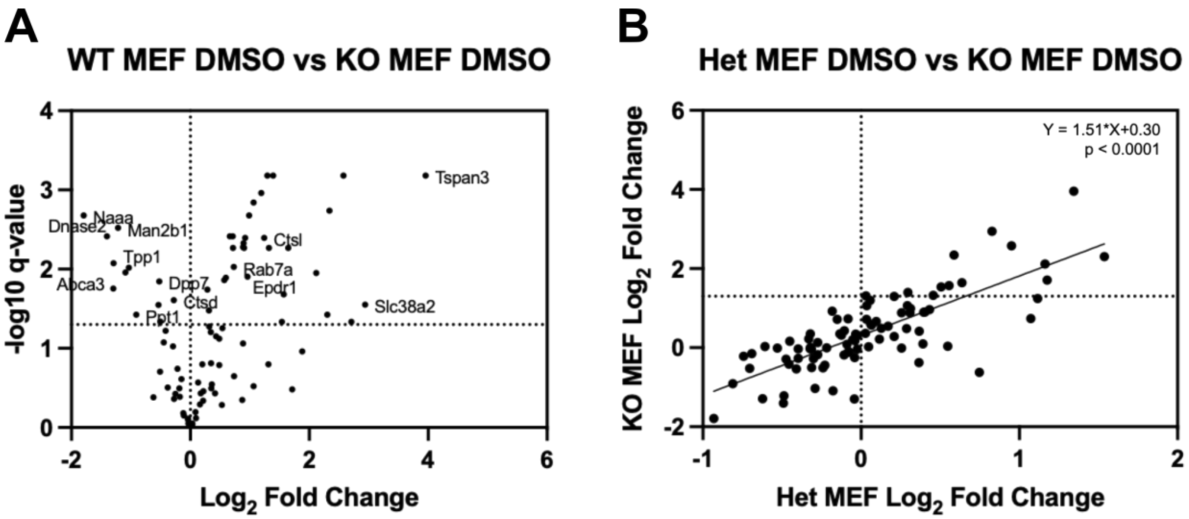

Supplementary Figure 8. *Grn*$^{-/-}$ MEF lysosomal proteomes are altered compared to *Grn*$^{+/+}$ lysosomes. (A) Volcano plots of fold changes versus *Grn*$^{+/+}$ controls of *Grn*$^{-/-}$ MEF LysoIP isolated proteomes (n=6 *Grn*$^{+/+}$, n=*6 Grn*$^{-/-}$)(multiple t-tests followed by two-stage step-up Benjamini, Krieger, and Yekutieli test). **(B)** Proteins analyzed in both *Grn*$^{+/-}$ and *Grn*$^{-/-}$ LysoIP TMT-MS experiments demonstrate correlated fold changes compared to *Grn*$^{+/+}$ lysosomes (****p<0.0001, simple linear regression).

Caco-2 cell Permeability Papp Value (10e^{-6} cm/s)					
Treatment	Blank (no cells)		Caco-2 cells		Efflux Ratio
	Papp A→B	Papp B→A	Papp A→B	Papp B→A	
C40	29.3	48.8	19.5	19.5	1.00
C127	37	37	3.7	5.57	1.51
Propranolol	51.5	51.5	25.7	25.7	1.00
Naldolol	61.3	61.3	0.22	1.23	5.59
Quinidine	42.8	42.8	10.7	42.8	4.00
Cimetidine	49.7	49.7	0.5	4.9	9.80

Supplementary Table 1. In vitro assessment permeability using Caco-2 cells. No transporter interaction demonstrated if efflux ratio is <2. Propranolol, naldolol, quinidine and cimetidine were controls for Caco-2 function.
