# Supplementary material for: Benzoxazole-derivatives enhance progranulin expression and reverse the aberrant lysosomal proteome caused by GRN haploinsufficiency
Source: Nat Commun. 2024 Jul 20;15:6125. doi: 10.1038/s41467-024-50076-8 (PMC11271458; doi:10.1038/s41467-024-50076-8)
Supplement: Supplementary file 3 — Description of Additional Supplementary Files [file 41467_2024_50076_MOESM3_ESM.pdf]

Description of Additional Supplementary Files

Supplementary Data 1. Molecular structures of tested compounds including catalog numbers.

Supplementary Data 2. NMR and HRMS spectra for tested compounds.

Supplementary Data 3-9. TMT-MS analysis for MEF LysoIP (Supplementary Data 3-4), HDF LysoIP (Supplementary Data 5), HDF whole cell lysate (Supplementary Data 6), MEF+A41 LysoIP (Supplementary Data 7) and A41 treated HDF LysoIP (Supplementary Data 8-9). Fold changes of protein abundances detected in TMT-MS experiments. Supplementary Data 3-4 compares lysosome proteomes between Grn+/+, Grn+/-, and Grn-/- MEFs. Supplementary Data 5 compares lysosome proteomes between healthy and GRN-FTD patient HDFs. Supplementary Data 6 compares whole cell proteomes between healthy and GRN-FTD patient HDFs. Supplementary Data 7 compares lysosome proteomes between DMSO treated Grn+/+, DMSO treated Grn+/-, and A41 treated Grn+/- MEFs. Supplementary Data 8-9 compares lysosome proteomes between DMSO treated healthy patient HDFs, DMSO treated GRN-FTD patient HDFs, and A41 treated GRN-FTD patient HDF.
