# Supplementary material for: Benzoxazole-derivatives enhance progranulin expression and reverse the aberrant lysosomal proteome caused by GRN haploinsufficiency
Source: Nat Commun. 2024 Jul 20;15:6125. doi: 10.1038/s41467-024-50076-8 (PMC11271458; doi:10.1038/s41467-024-50076-8)
Supplement: Supplementary file 4 — Supplementary Data 1 [file 41467_2024_50076_MOESM4_ESM.pdf]

Compound #		MW	Catalog #	Company	
C1	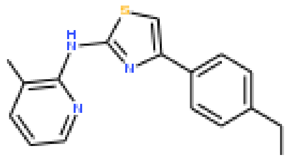	295.4	5186-0091	ChemDiv	
C2	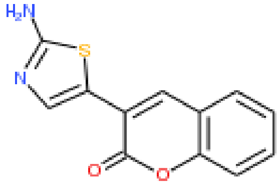	244.274	0833-0689	ChemDiv	
C3	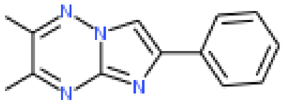	224.265	8010-5751	ChemDiv	
C4	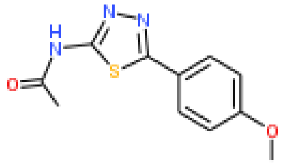	249.293	3388-0199	ChemDiv	
C5	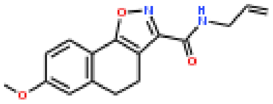	284.30984	C226-4038	ChemDiv	

C6	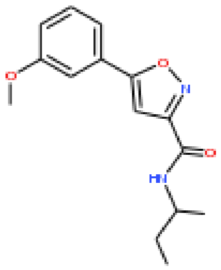	274.31502	C226-0835	ChemDiv	
C7	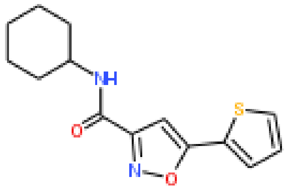	276.359	C226-1835	ChemDiv	
C8	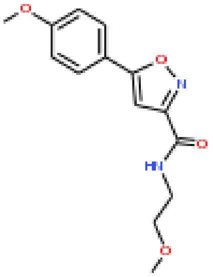	276.28784	C226-0715	ChemDiv	
C9	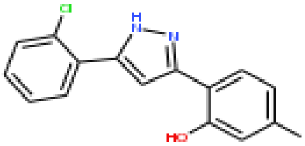	*285*	5481021	Chembridge	
C10	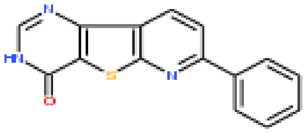	279.32	5285006	Chembridge	

C11	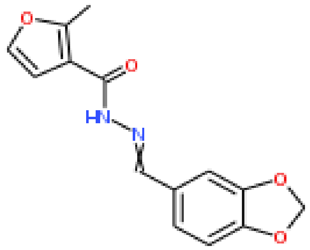	272.26	5472332	Chembridge	
C12	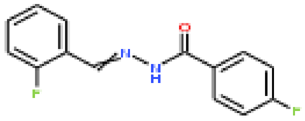	260.24	5374236	Chembridge	
C13	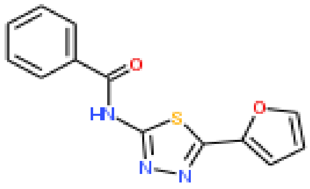	271.29	5630898	Chembridge	
C14	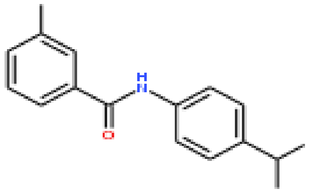	253.34	5257208	Chembridge	
C15	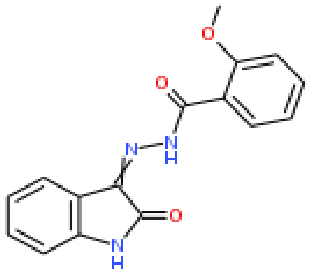	295.29	5185123	Chembridge	

C16	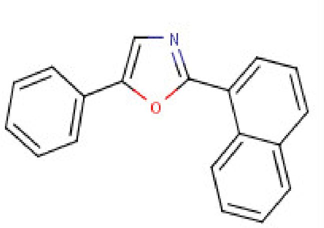	*271*	7617105	Chembridge	
C17	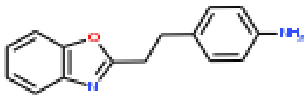	238.28	0717-0951	Chemdiv	
C18	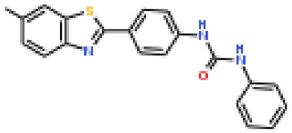	359.44	4768-0456	Chemdiv	
C19	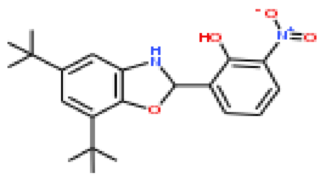	370.44	8009-7313	Chemdiv	
C20	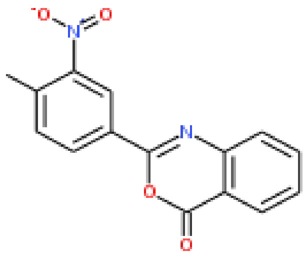	282.25	0591-5672	Chemdiv	

C21	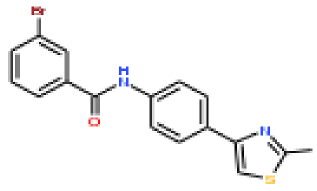	373.27	3384-8159	Chemdiv	
C22	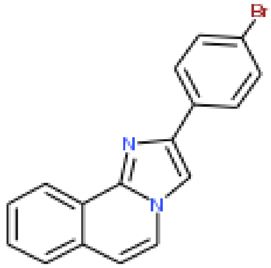	323.19	3312-0097	Chemdiv	
C23	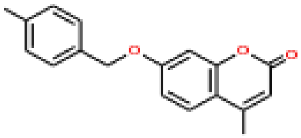	280.32	3330-4106	Chemdiv	
C24	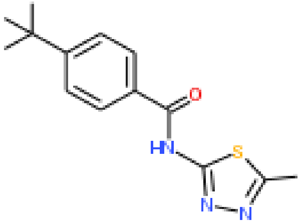	275.37	2237-0111	Chemdiv	
C25	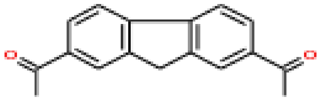	250.3	5107566	Chembridge	

C26	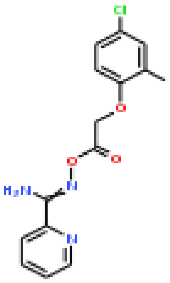	319.7	5800256	Chembridge	
C27	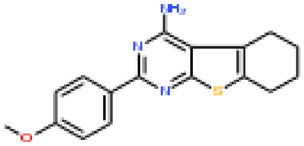	311.4	7392321	Chembridge	
C28	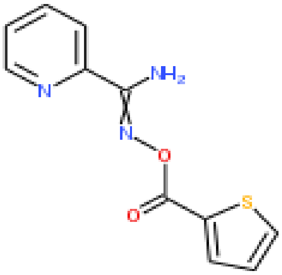	247.3	5651966	Chembridge	
C29	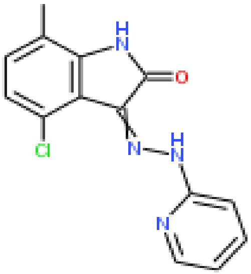	286.7	5623079	Chembridge	
C30	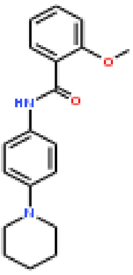	310.4	6317545	Chembridge	

C31	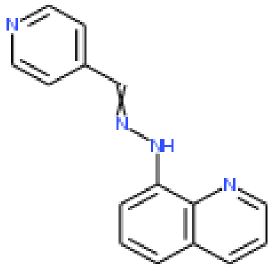	248.3	5618390	Chembridge	
C32	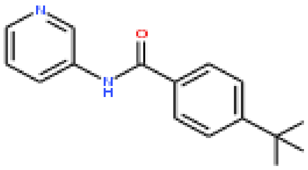	254.3	5650272	Chembridge	
C33	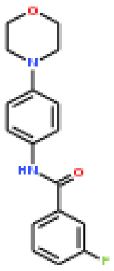	300.3	6321437	Chembridge	
C34	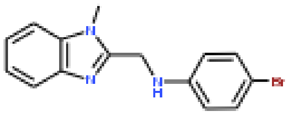	254.3	5648872	Chembridge	
C35	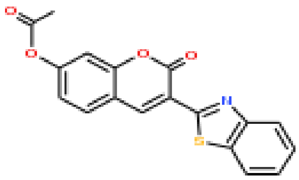	337.4	5554608	Chembridge	

C36	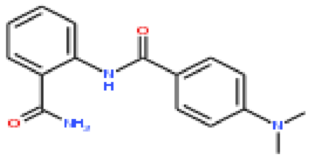	283.3	6100050	Chembridge	
C37	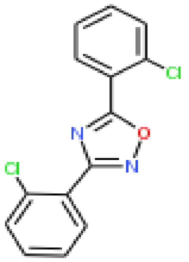	291.1	5785642	Chembridge	
C38	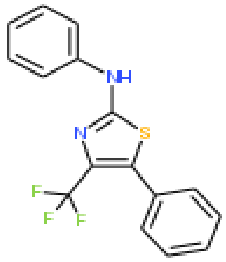	320.3	5185546	Chembridge	
C39	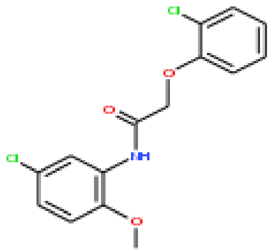	326.2	5801675	Chembridge	
C40	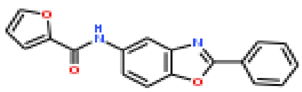	304.3	6328802	Chembridge	

C41	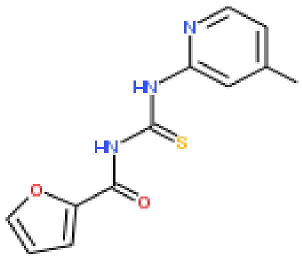	261.3	6161539	Chembridge	
C42	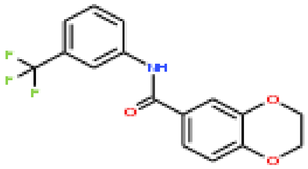	323.3	5790356	Chembridge	
C43	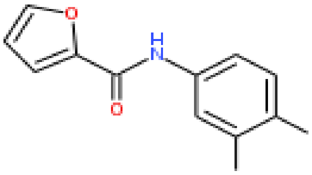	215.3	5246370	Chembridge	
C44	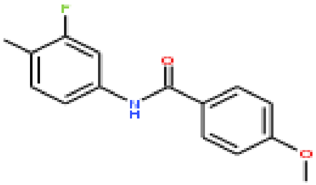	259.3	6451668	Chembridge	
C45	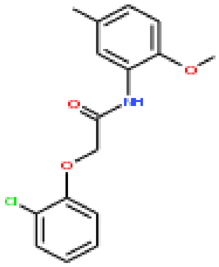	305.8	5802434	Chembridge	

C46	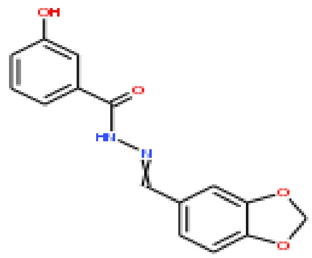	284.3	5132321	Chembridge	
C47	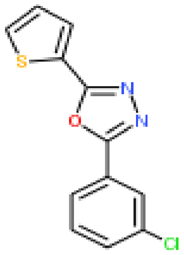	262.7	5400917	Chembridge	
C48	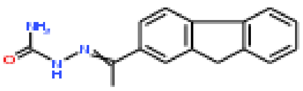	265.3	5121891	Chembridge	
C49	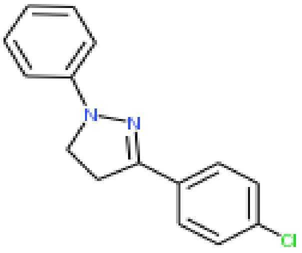	256.7	5255935	Chembridge	
C50	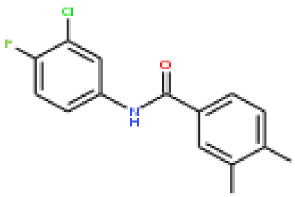	277.7	5648978	Chembridge	

C51	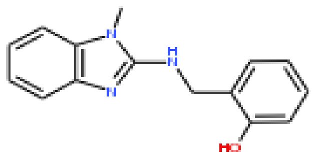	253.3	6511960	Chembridge	
C52	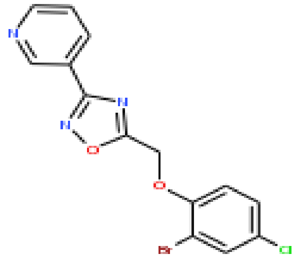	366.6	5802987	Chembridge	
C23	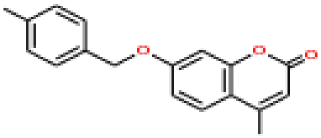	280.32	3330-4106	Chemdiv	
C24	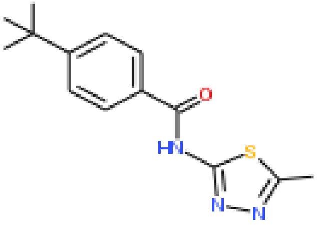	275.37	2237-0111	Chemdiv	
C53	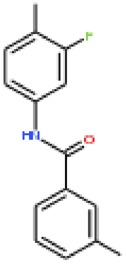	243.28	1699-1205	Chemdiv	

C54	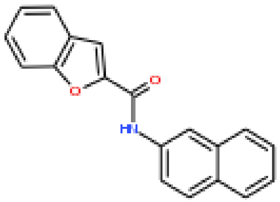	287.31	2365-0448	Chemdiv	
C55	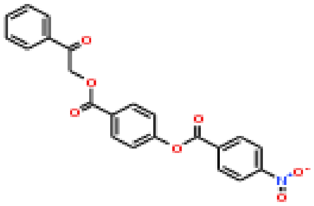	405.36	8006-3398	Chemdiv	
C56	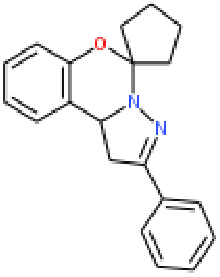	304.39	0443-0006	Chemdiv	
C57	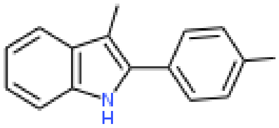	221.3	8007-0879	Chemdiv	
C58	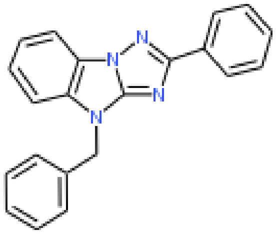	324.38	6325-0272	Chemdiv	

C59	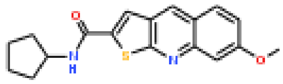	326.41	C303-0453	Chemdiv	
C60	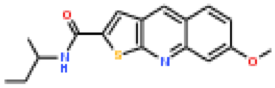	314.4	C303-0266	Chemdiv	
C61	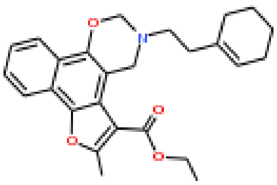	419.51	4550-6223	Chemdiv	
C62	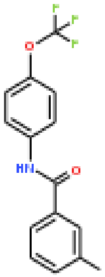	295.26	1699-1201	Chemdiv	
C63	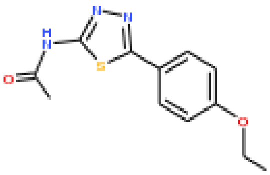	263.32	8010-2154	Chemdiv	

C64	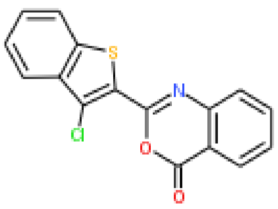	313.76	1982-0366	Chemdiv	
C65	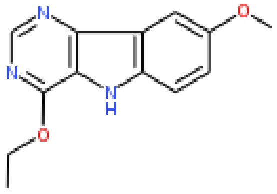	243.26	4138-0078	Chemdiv	
C66	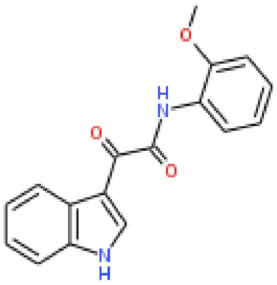	294.3	6143-0066	Chemdiv	
C67	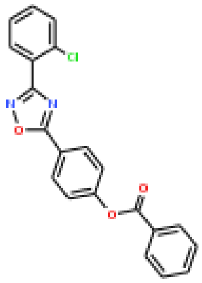	376.79	K086-0092	Chemdiv	
C68	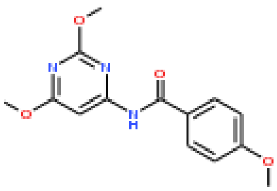	289.29	8010-2955	Chemdiv	

C69	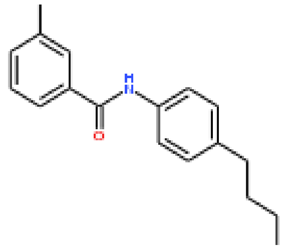	267.37	1683-5579	Chemdiv	
C70	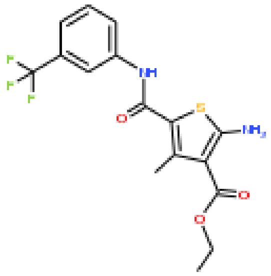	372.36	2496-3892	Chemdiv	
C71	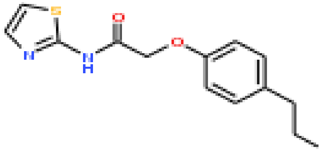	276.35	1683-7020	Chemdiv	
C72	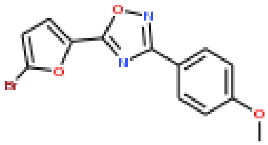	321.13	5631-0983	Chemdiv	
C73	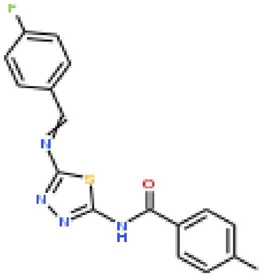	340.38	0663-1037	Chemdiv	

C74	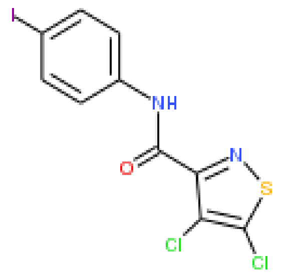	399.04	4872-0345	Chemdiv	
C75	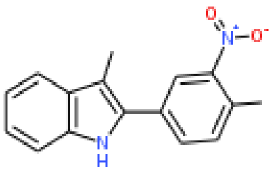	266.29	8007-1121	Chemdiv	
C76	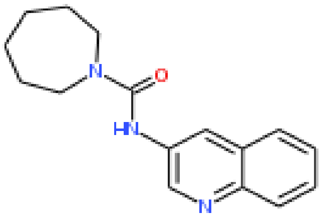	269.34	5582-0085	Chemdiv	
C77	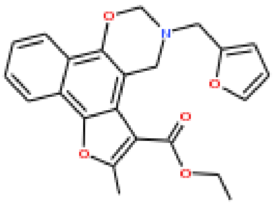	391.42	4552-3263	Chemdiv	
C78	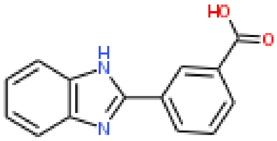	238.24	4587-0392	Chemdiv	

C79	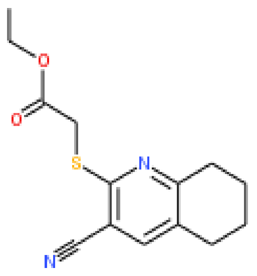	276.35	3419-0101	Chemdiv	
C80	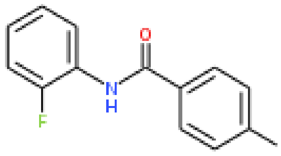	229.25	2022-0634	Chemdiv	
C81	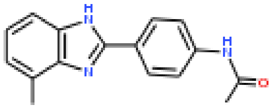	265.31	0375-0461	Chemdiv	
C82	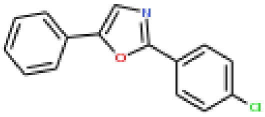	255.7	3531-5278	Chemdiv	
C83	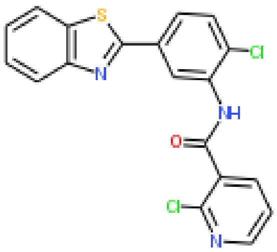	400.28	4376-0163	Chemdiv	

C84	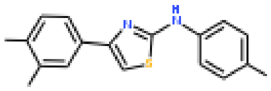	294.41	8007-4013	Chemdiv	
C85	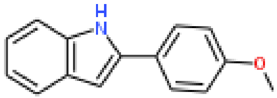	223.27	6292-0266	Chemdiv	
C86	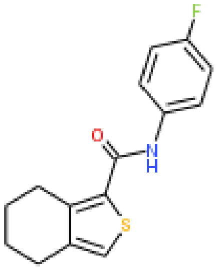	275.34	8012-7747	Chemdiv	
C87	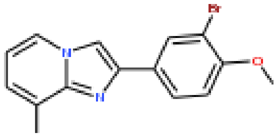	317.18	4341-2750	Chemdiv	
C88	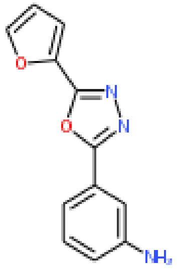	227.22	3952-2241	Chemdiv	

C89	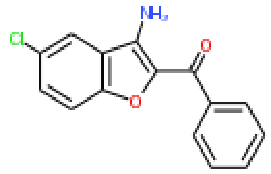	271.7	8010-5787	Chemdiv	
C90	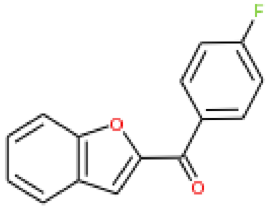	240.23	6220-7486	Chemdiv	
C91	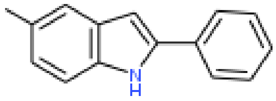	207.27	8005-4486	Chemdiv	
C92	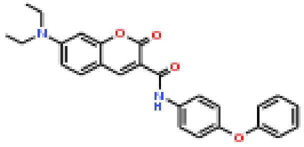	428.48	K219-1059	Chemdiv	
C93	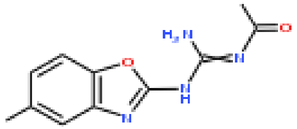	232.24	3456-3896	Chemdiv	

C94	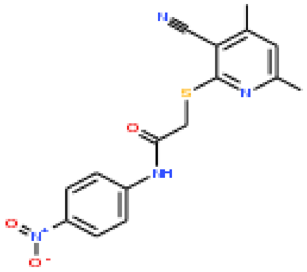	342.37	4486-0135	Chemdiv	
C95	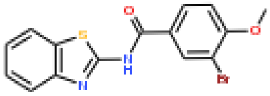	363.23	3617-1625	Chemdiv	
C96	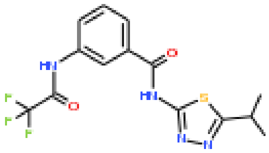	358.34	8006-9638	Chemdiv	
C97	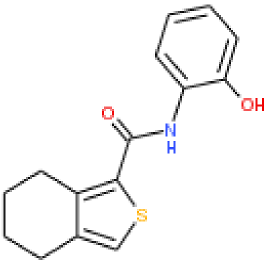	273.35	8012-4539	Chemdiv	
C98	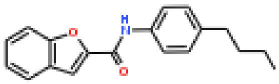	293.36	1988-1362	Chemdiv	

C99	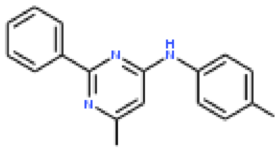	275.35	3752-0032	Chemdiv	
C100	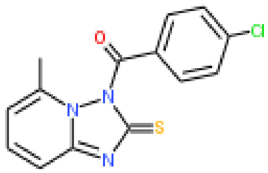	303.77	8013-1462	Chemdiv	
C101	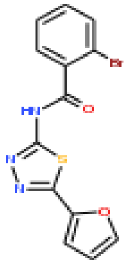	350.19	3388-0663	Chemdiv	
C102	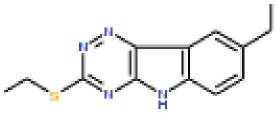	258.34	4896-2997	Chemdiv	
C103	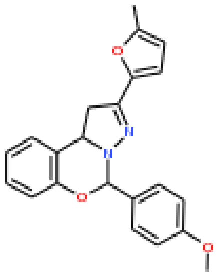	360.41	5793-0190	Chemdiv	

C104	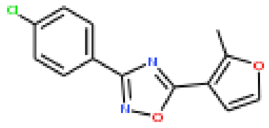	260.68	5631-0019	Chemdiv	
C105	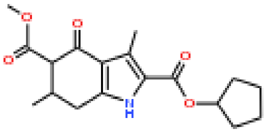	333.38	C276-0473	Chemdiv	
C106	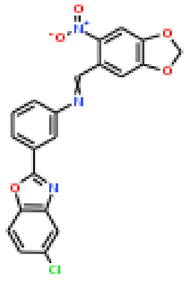	421.79	8005-6023	Chemdiv	
C107	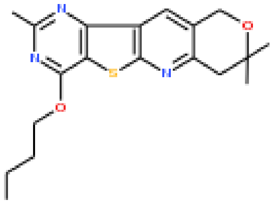	357.47	6131-0076	Chemdiv	
C108	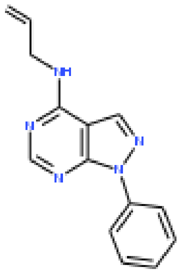	251.29	K402-0019	Chemdiv	

C109	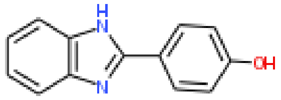	210.23	4385-2057	Chemdiv	
C110	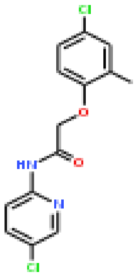	311.16	3617-0724	Chemdiv	
C111	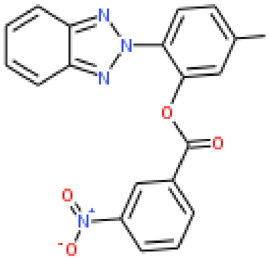	374.35	0584-0190	Chemdiv	
C112	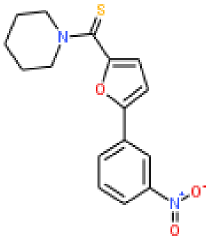	316.38	5143711	ChemBridge	
C113	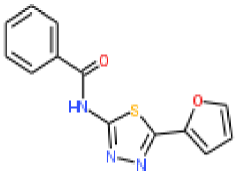	271.29	5630898	ChemBridge	

C114	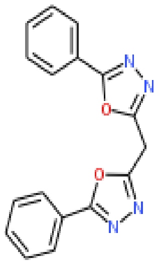	304.3	5223928	ChemBridge	
C115	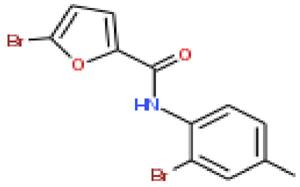	359.01	2043-4711	ChemDiv	
C116	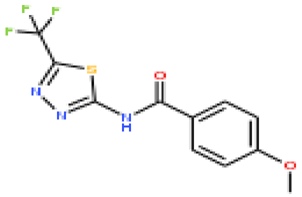	303.26	8005-2682	ChemDiv	
C117	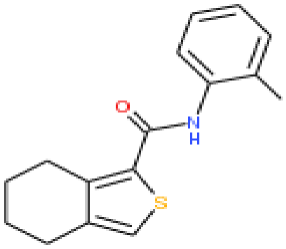	271.38	8012-4332	ChemDiv	
C118	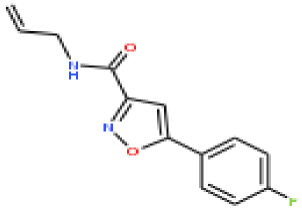	246.24	C226-0276	ChemDiv	

C119	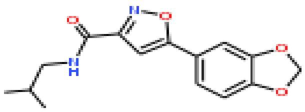	288.3	C226-1178	ChemDiv	
C120	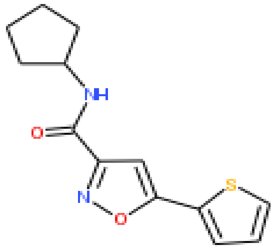	262.33	C226-1832	ChemDiv	
C121	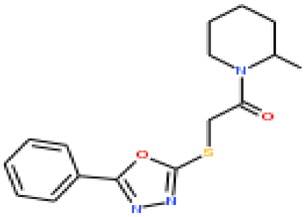	317.41	C292-0015	ChemDiv	
C122	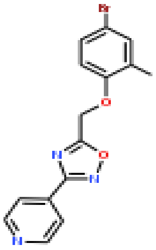	346.18	6319-3652	ChemDiv	
C123	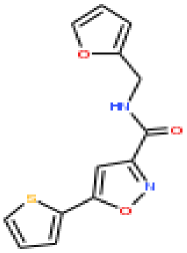	274.3	C226-1827	ChemDiv	

C124	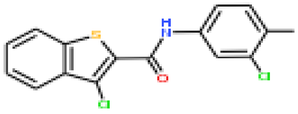	336.24	2043-2097	ChemDiv	
C125	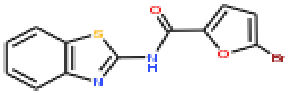	323.17	2042-2437	ChemDiv	
C126	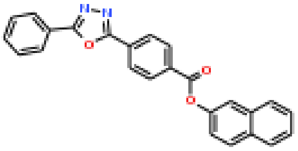	392.41	3257-3342	ChemDiv	
C127	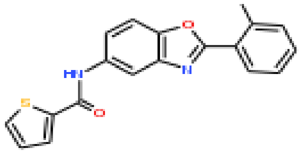	334.39	3864-1015	ChemDiv	
