# Supplementary material for: Benzoxazole-derivatives enhance progranulin expression and reverse the aberrant lysosomal proteome caused by GRN haploinsufficiency
Source: Nat Commun. 2024 Jul 20;15:6125. doi: 10.1038/s41467-024-50076-8 (PMC11271458; doi:10.1038/s41467-024-50076-8)

^{1}H-NMR of compound A01

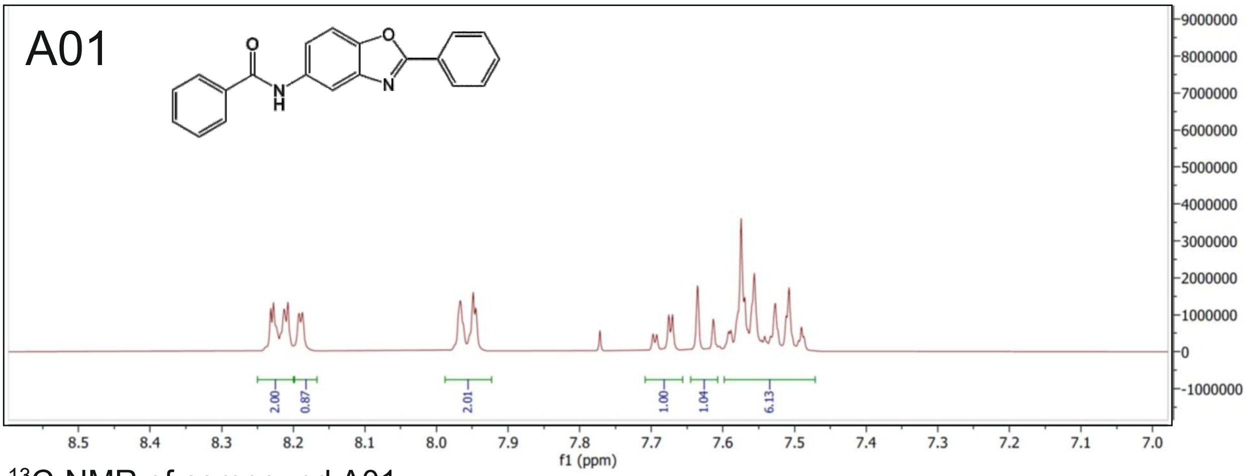

^{13}C-NMR of compound A01

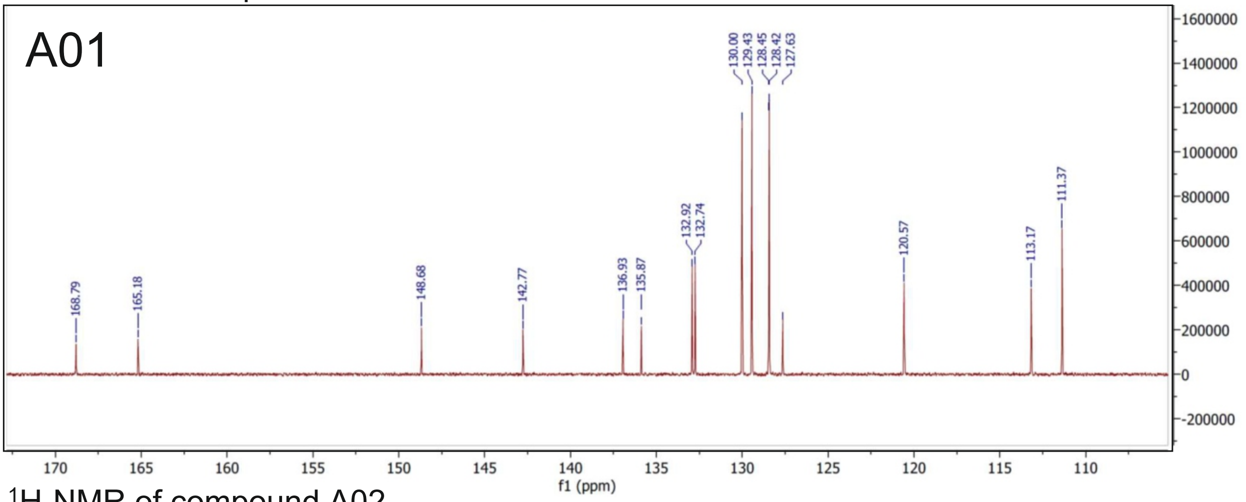

^{1}H-NMR of compound A02

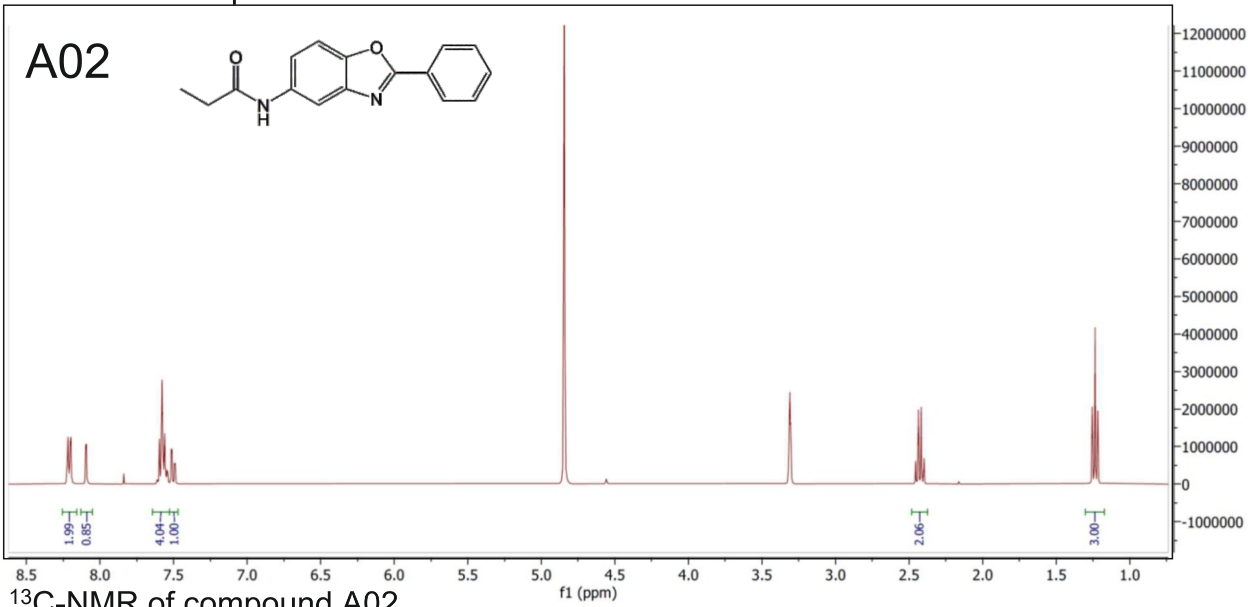

^{13}C-NMR of compound A02

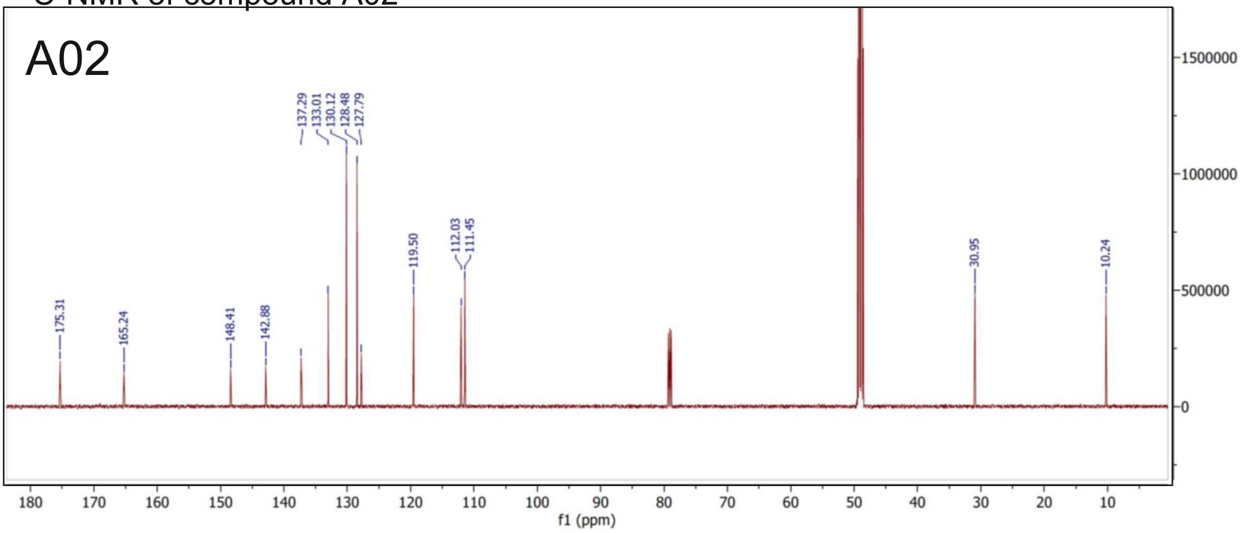

^{1}H-NMR of compound A03

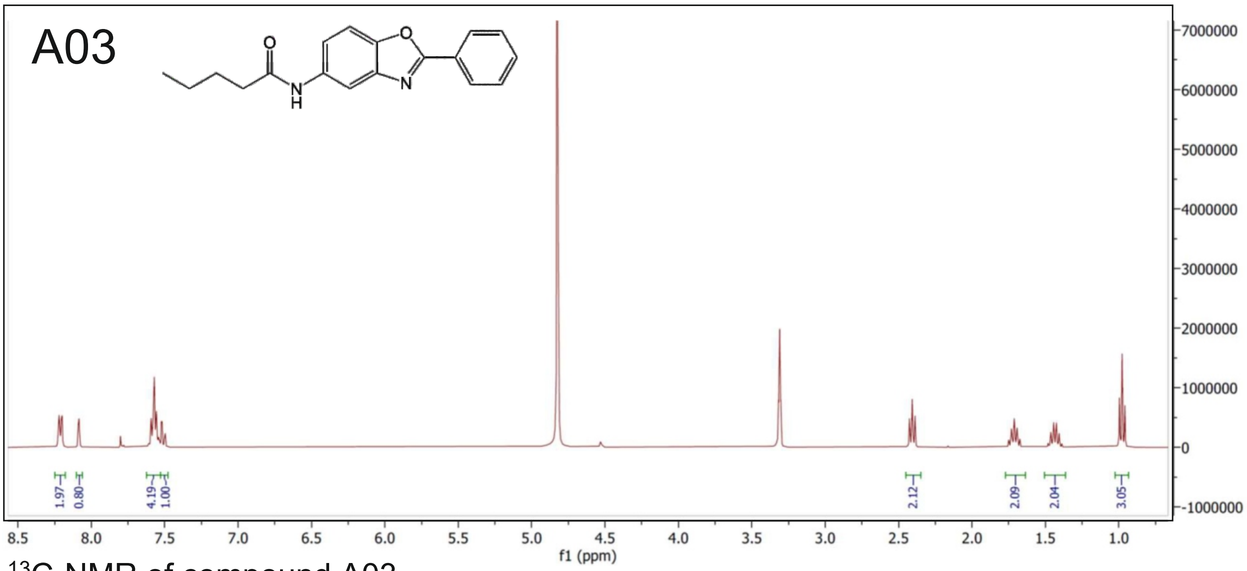

^{13}C-NMR of compound A03

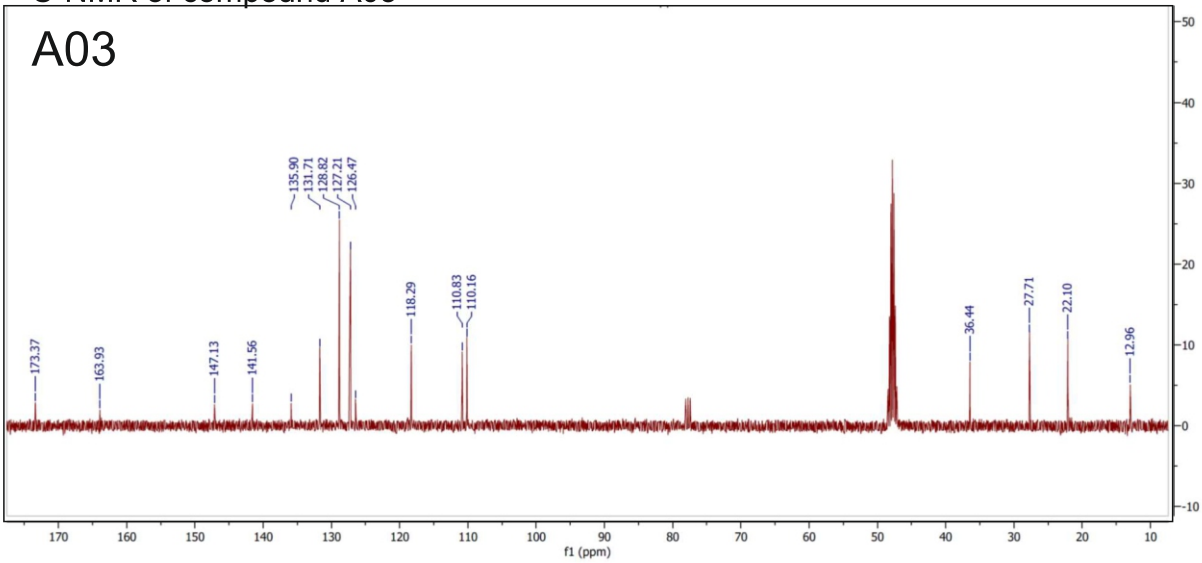

^{1}H-NMR of compound A04

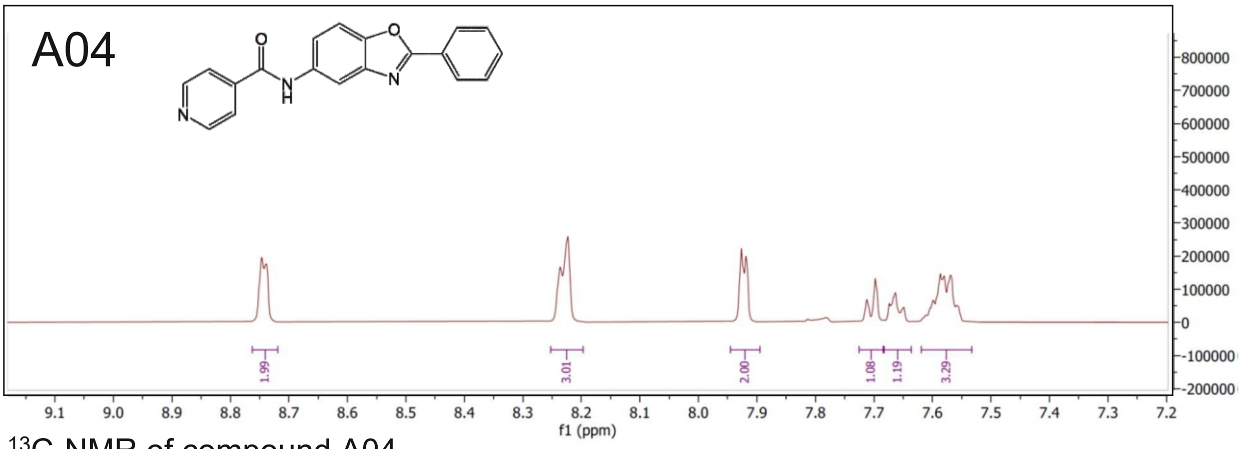

^{13}C-NMR of compound A04

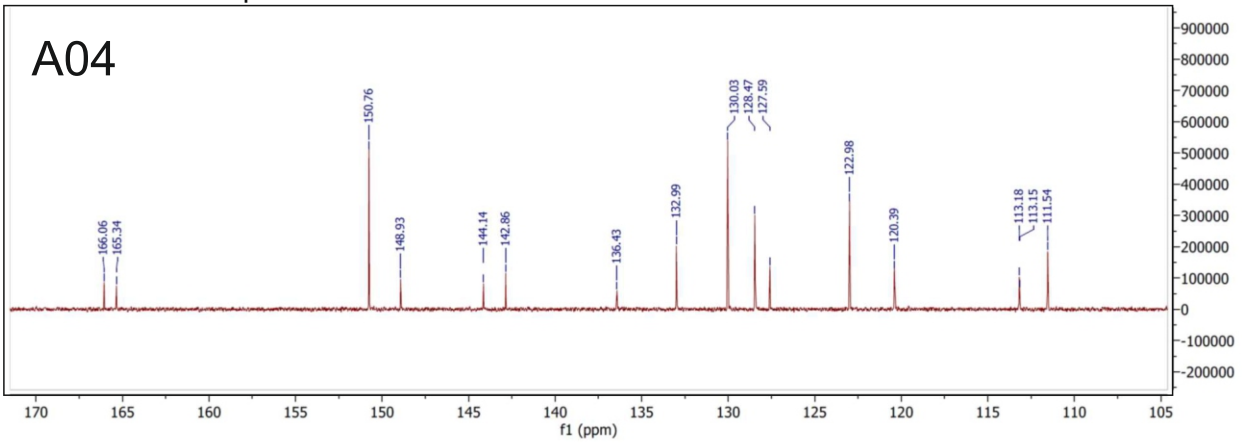

^{1}H-NMR of compound A05

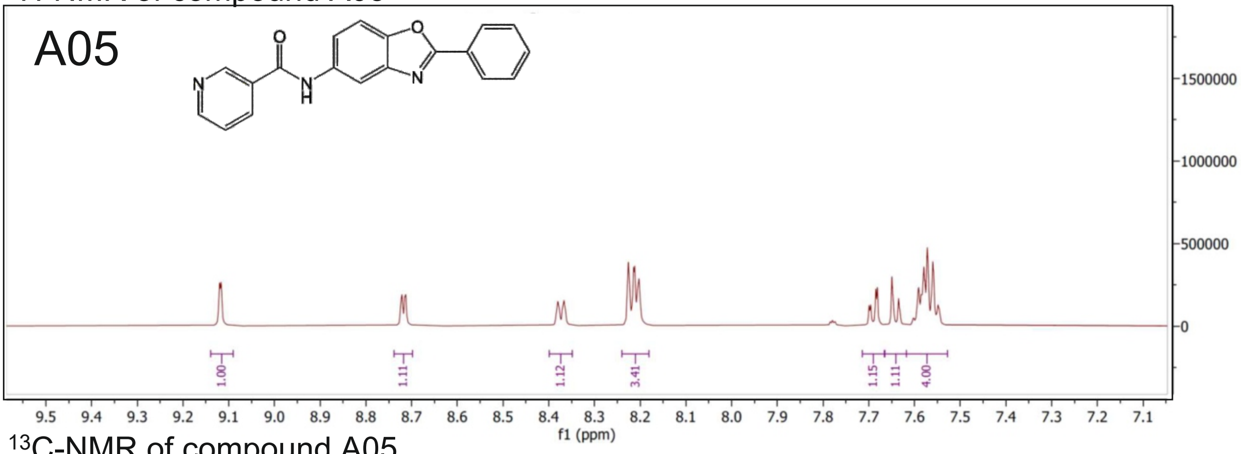

^{13}C-NMR of compound A05

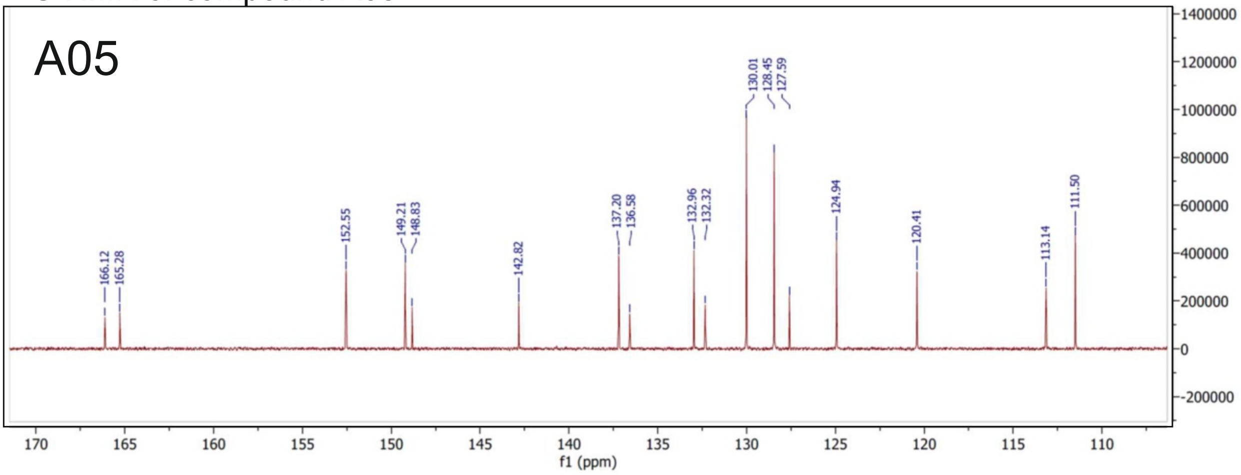

^{1}H-NMR of compound A06

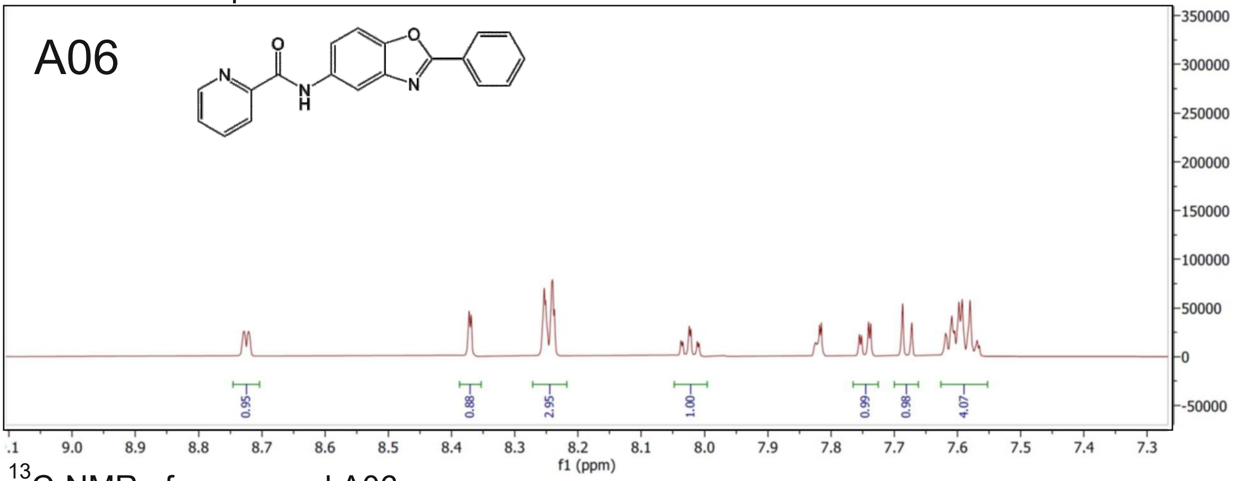

^{13}C-NMR of compound A06

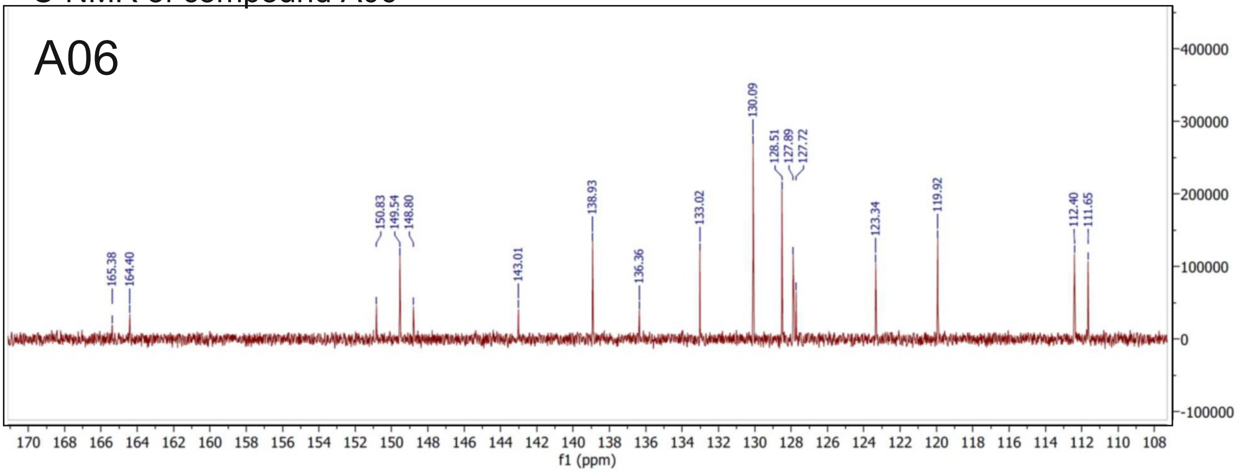

^{1}H-NMR of compound A07

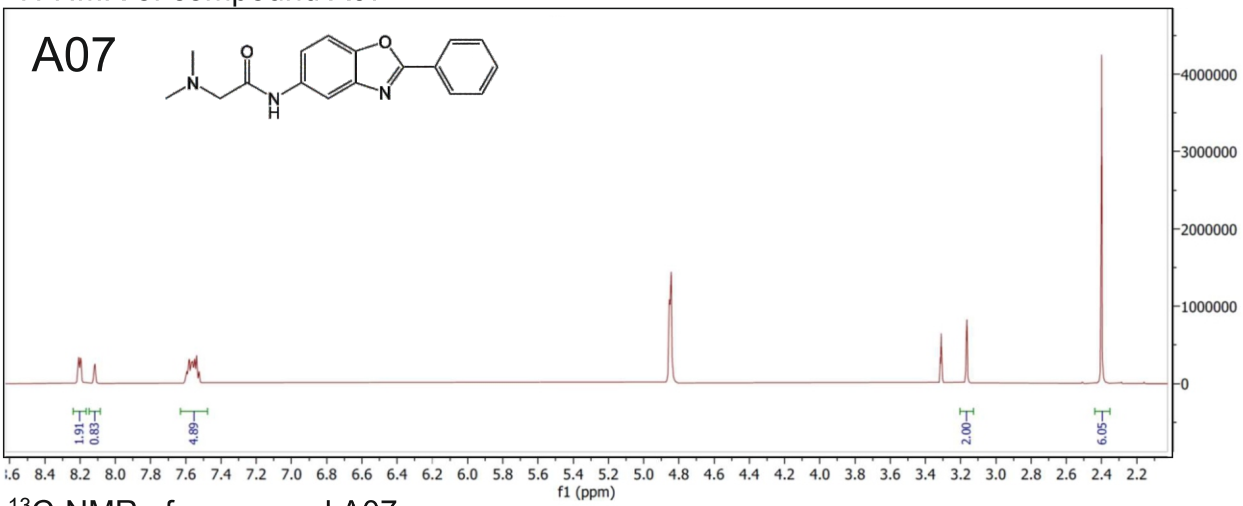

^{13}C-NMR of compound A07

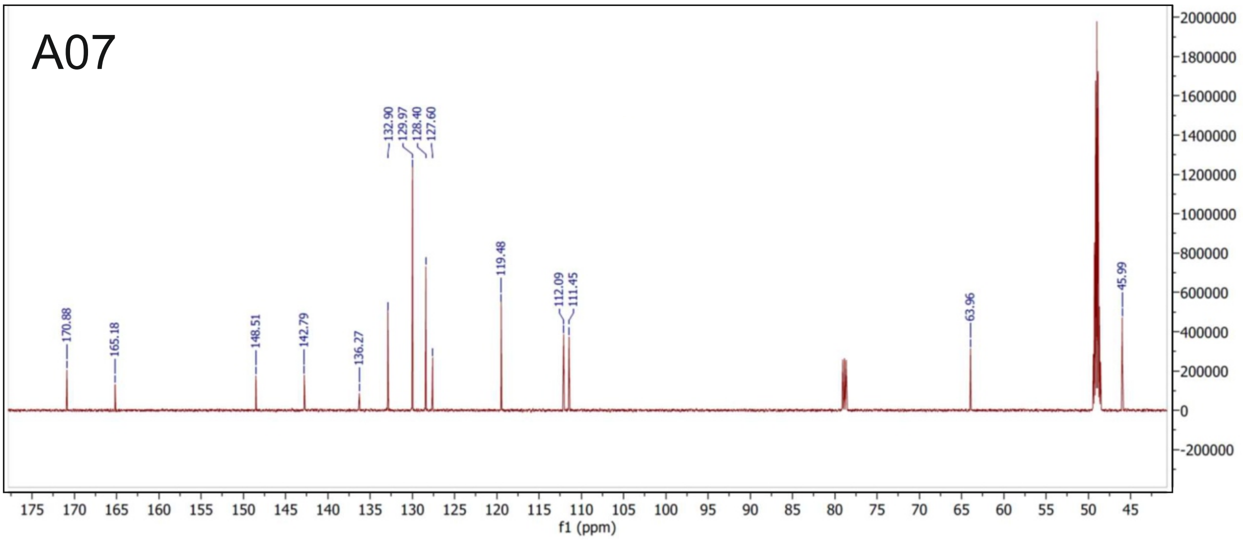

^{1}H-NMR of compound A08

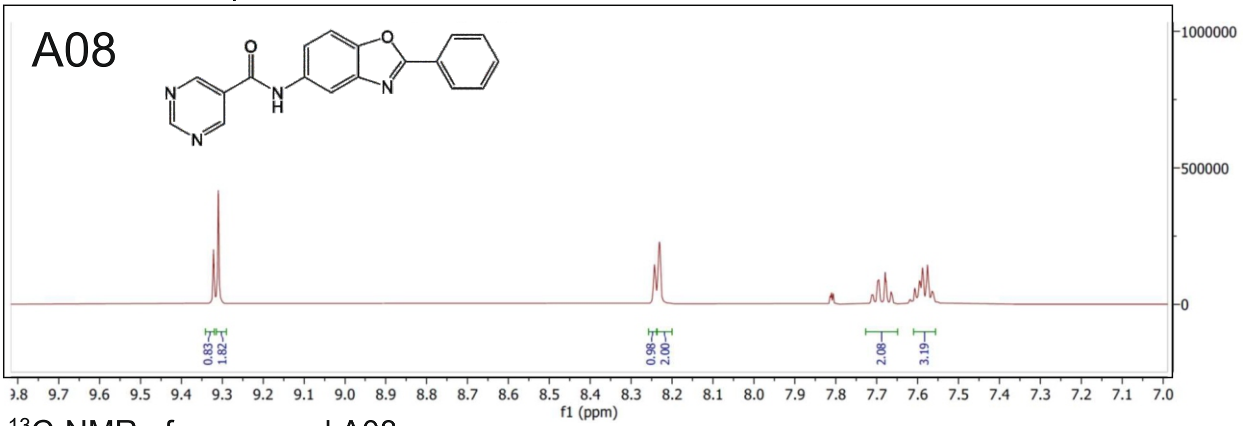

^{13}C-NMR of compound A08

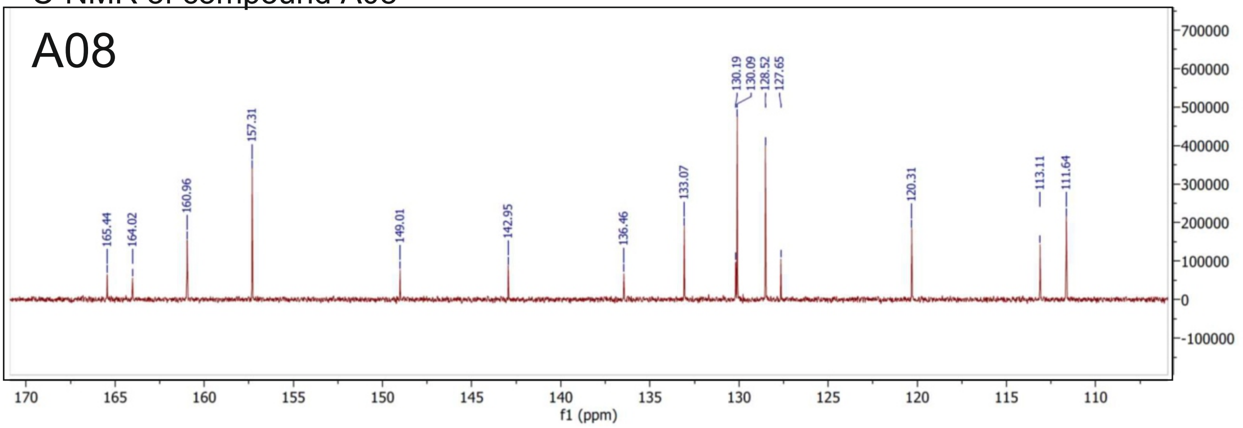

^{1}H-NMR of compound A09

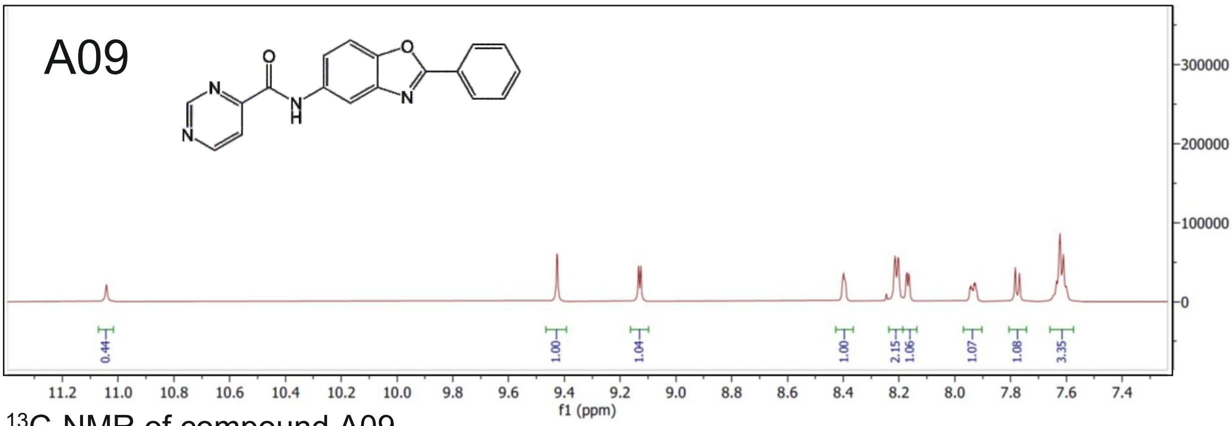

^{13}C-NMR of compound A09

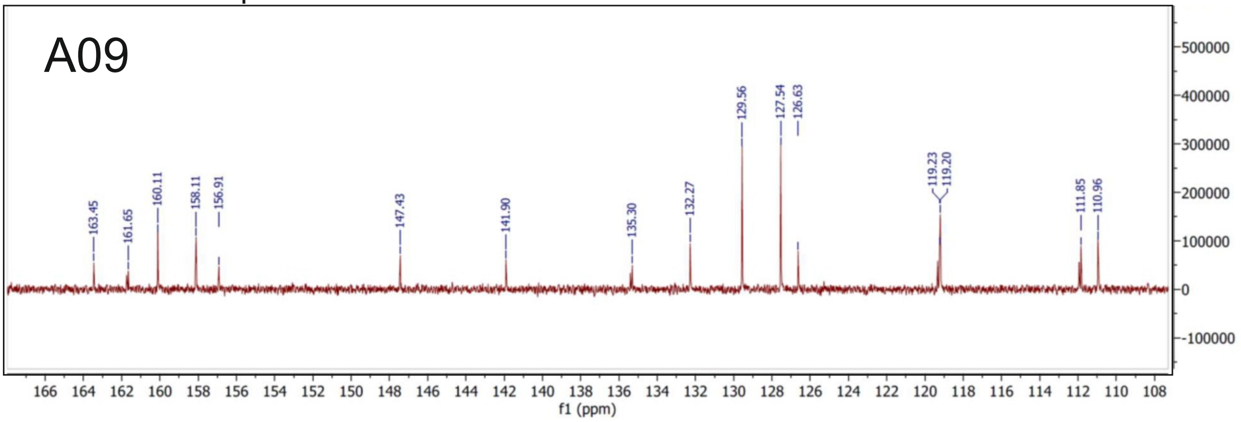

^{1}H-NMR of compound A10

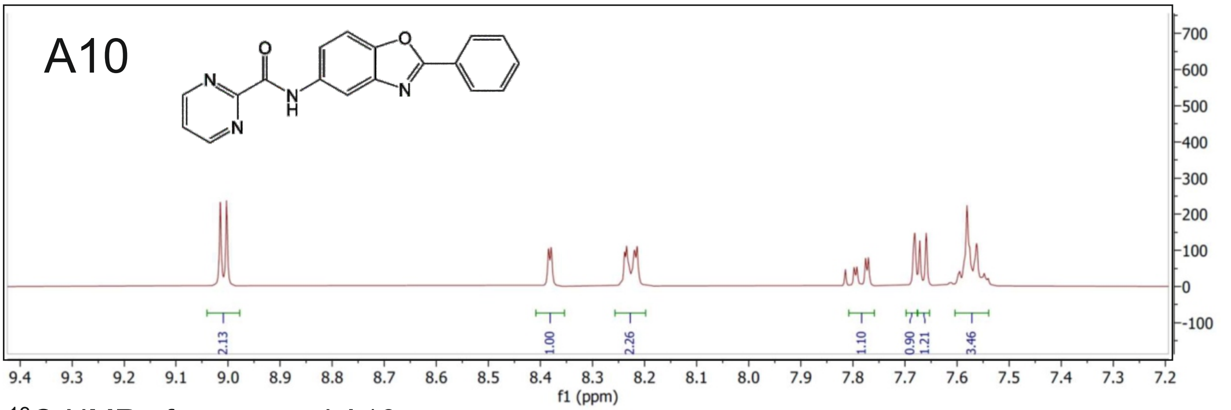

^{13}C-NMR of compound A10

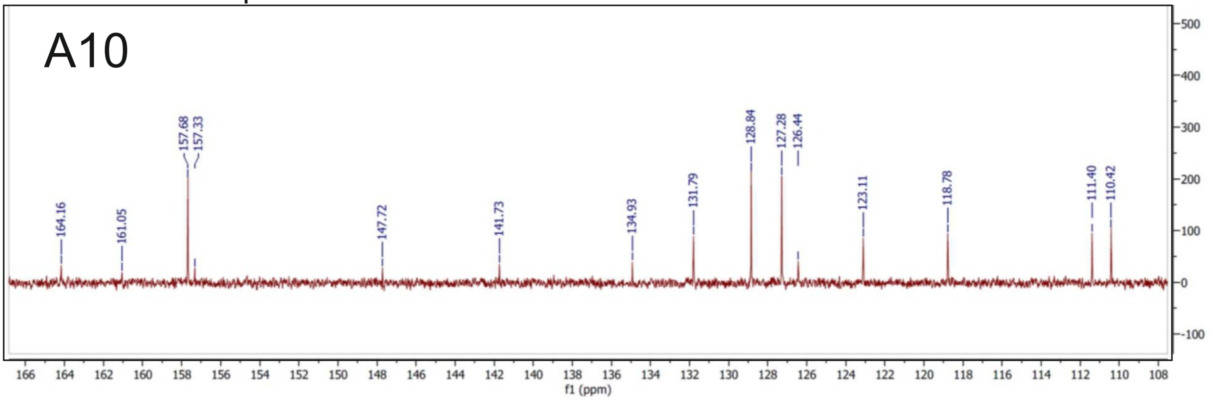

^{1}H-NMR of compound A11

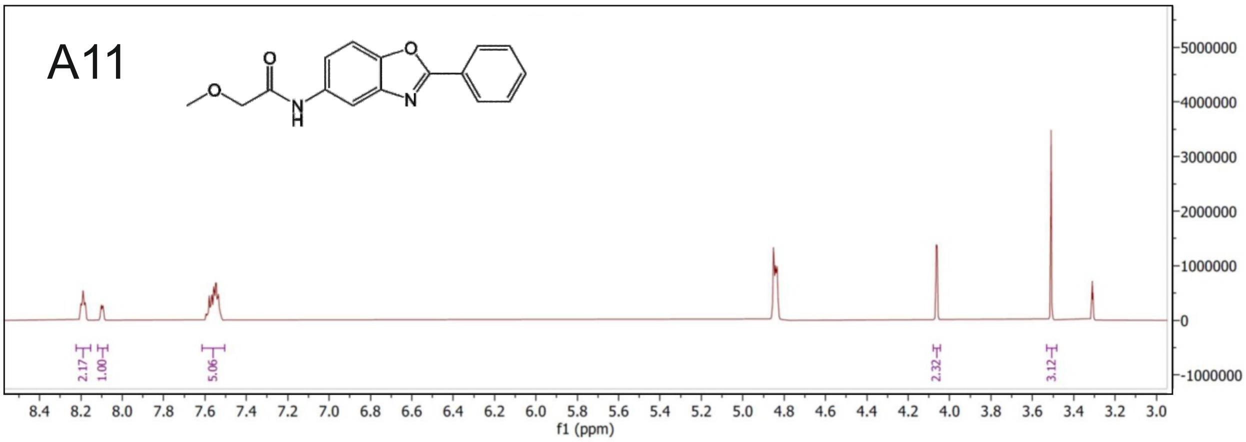

^{13}C-NMR of compound A11

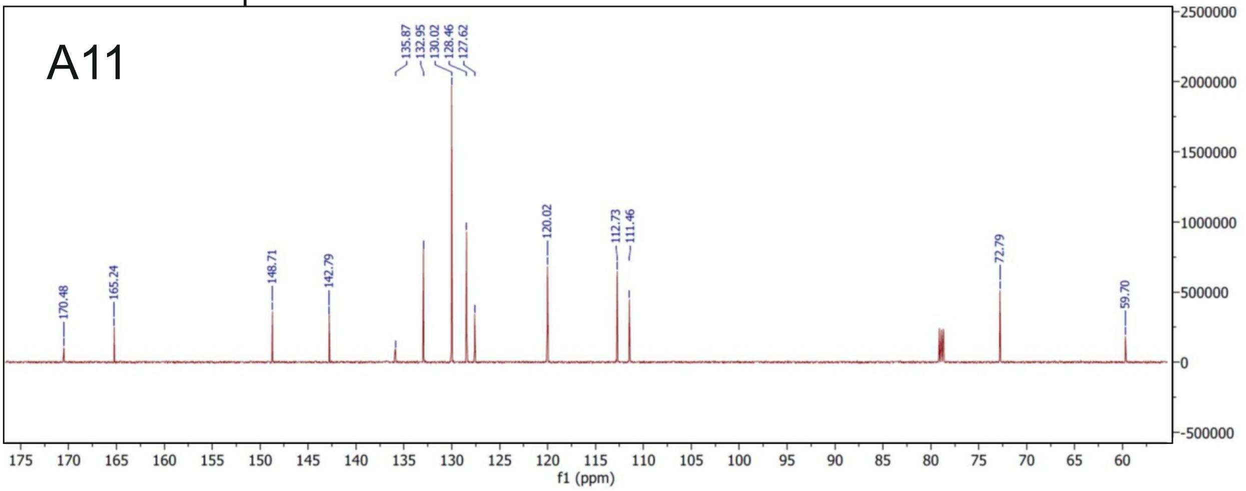

^{1}H-NMR of compound A12

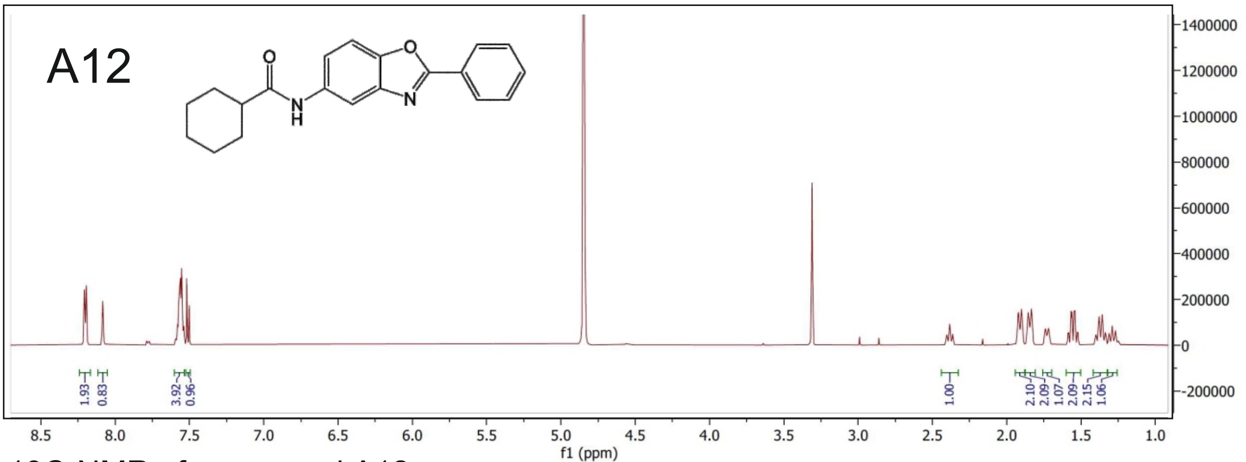

13C-NMR of compound A12

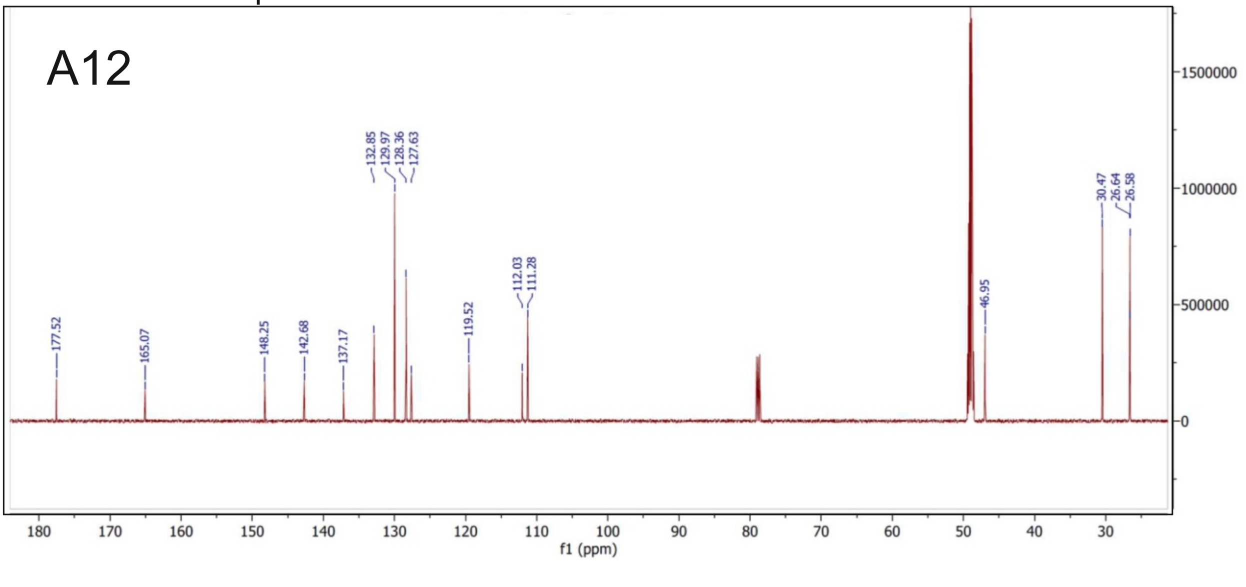

A16

^{1}H-NMR of compound A16

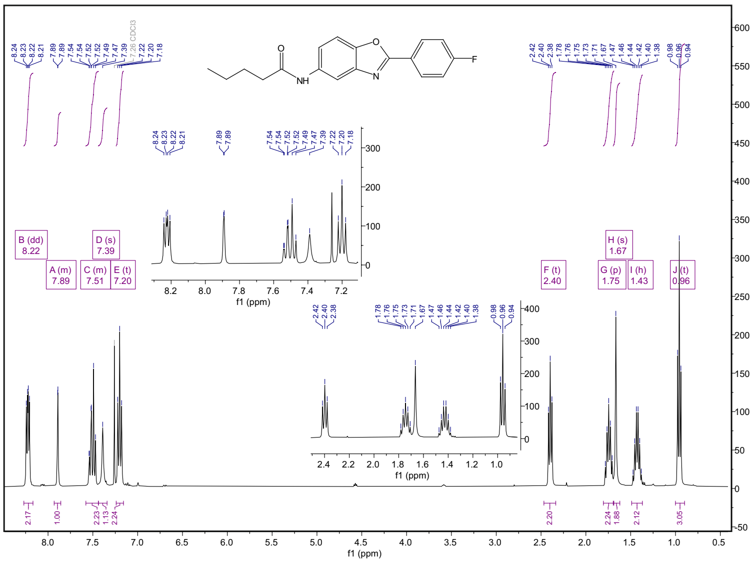

^{13}C-NMR of compound A16

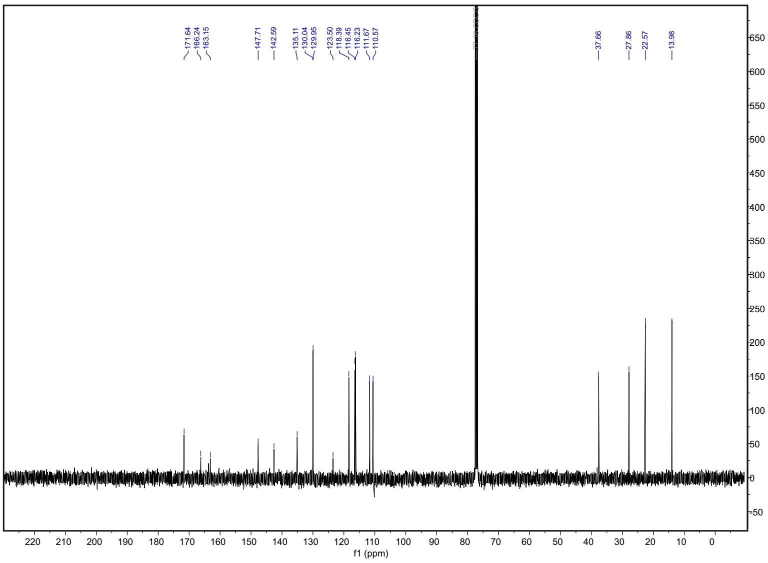

A17

^{1}H-NMR of compound A17

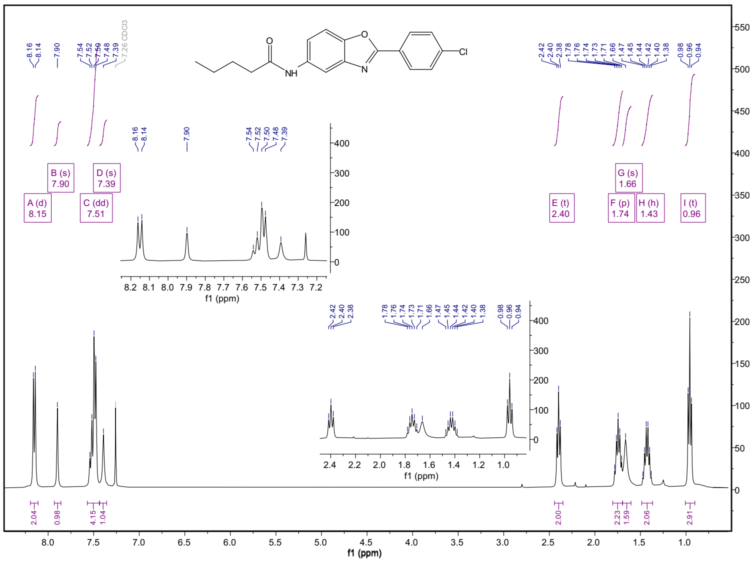

^{13}C-NMR of compound A17

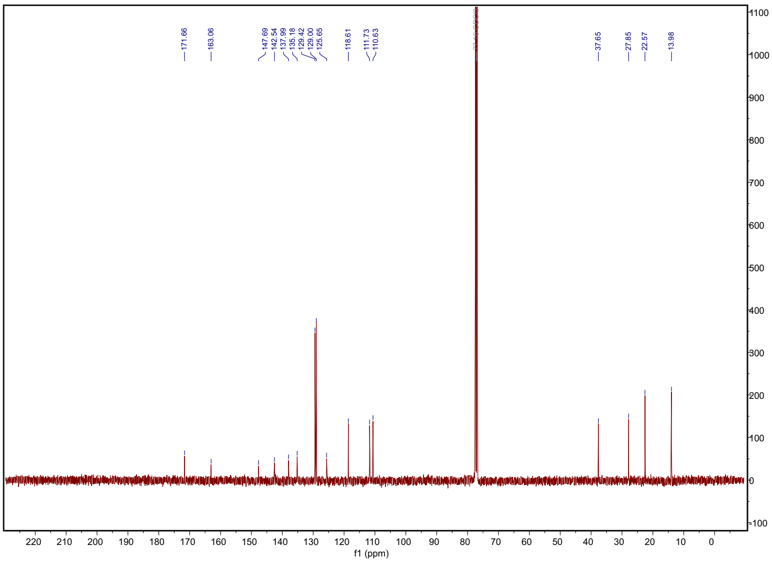

A20

^{1}H-NMR of compound A20

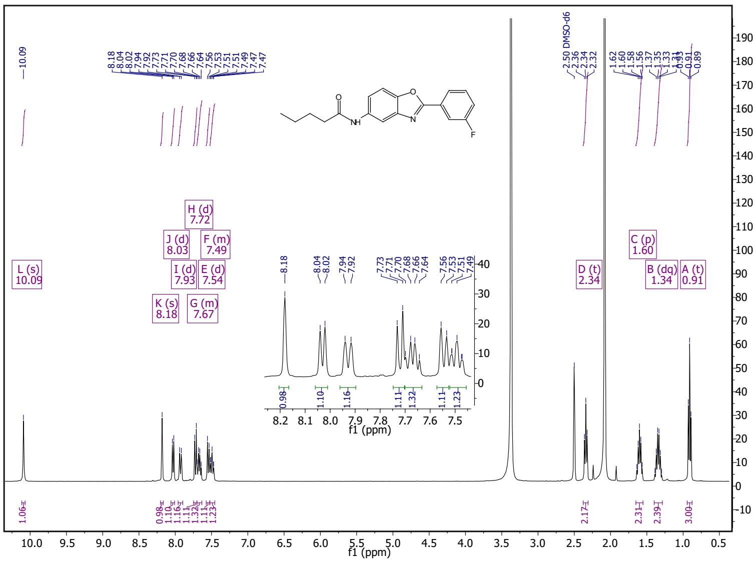

^{13}C-NMR of compound A20

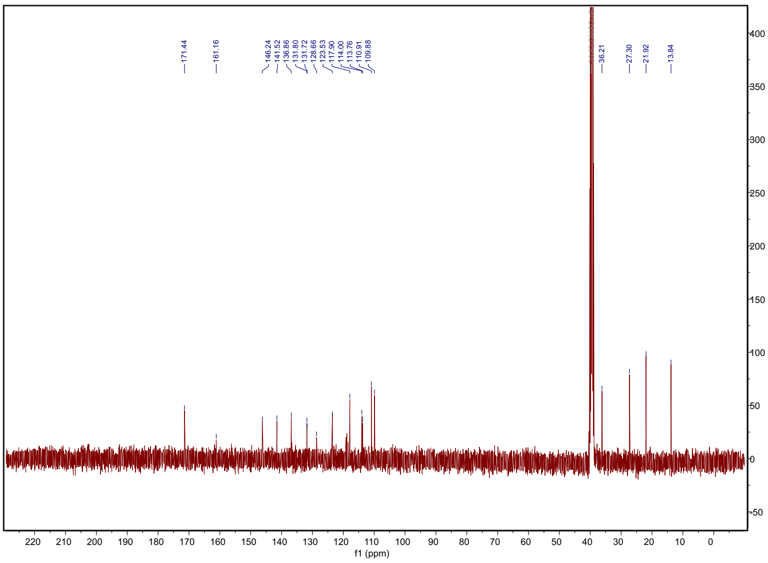

A21

^{1}H-NMR of compound A21

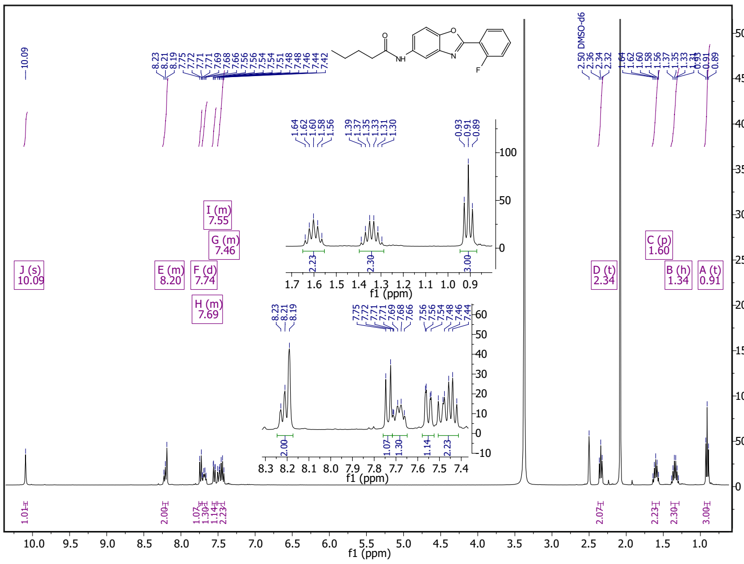

^{13}C-NMR of compound A21

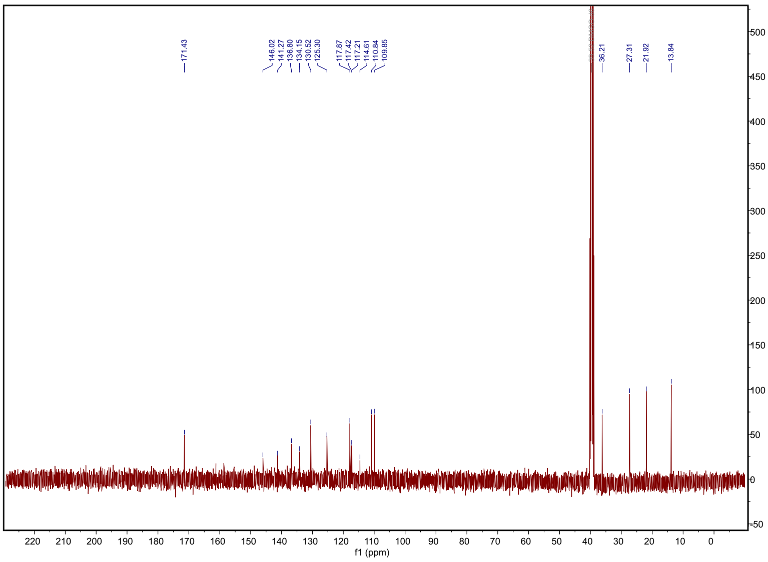

A22

^{1}H-NMR of compound A22

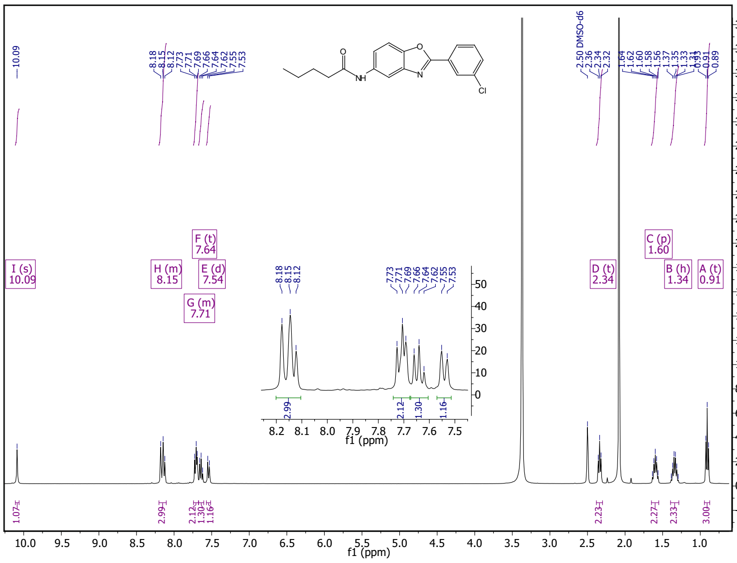

^{13}C-NMR of compound A22

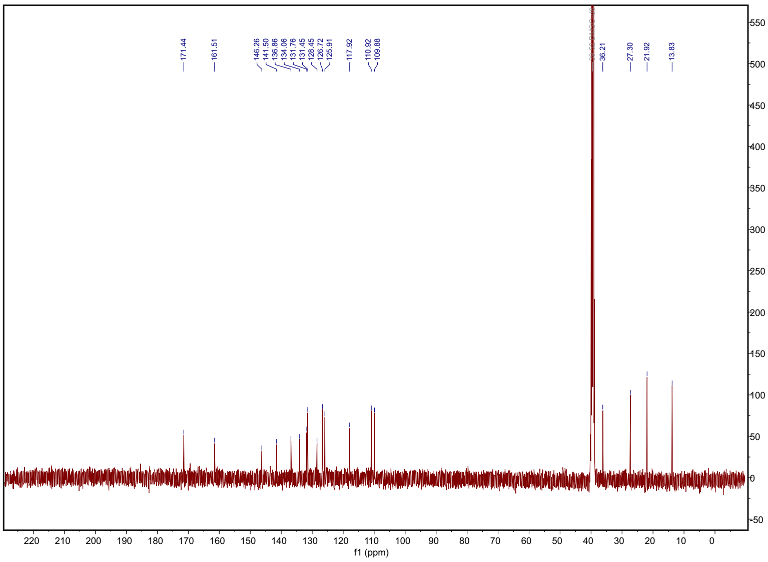

A24

^{1}H-NMR of compound A24

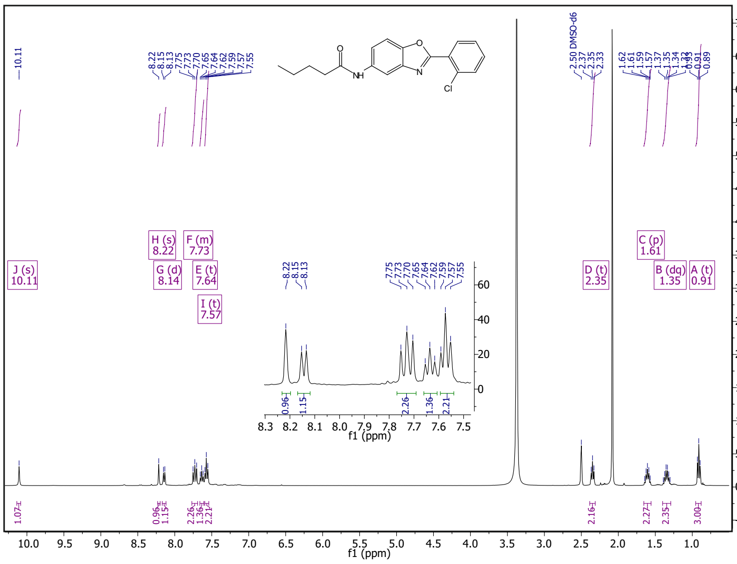

^{13}C-NMR of compound A24

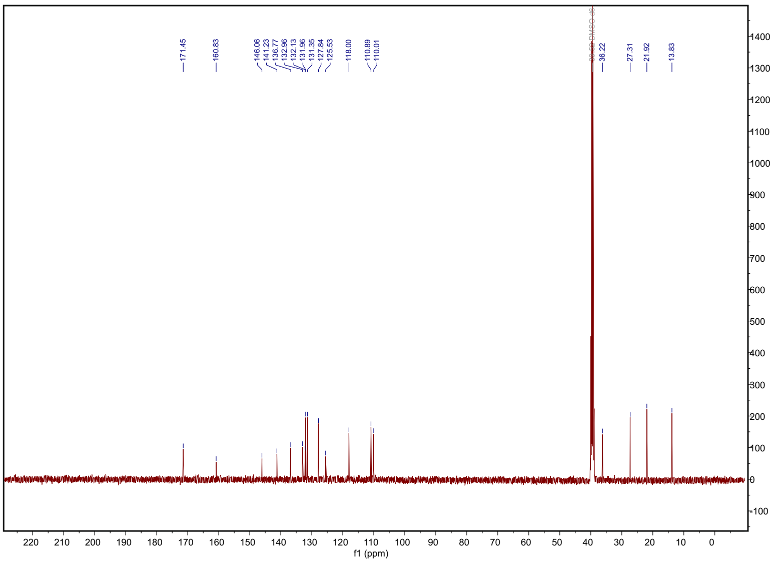

A28

^{1}H-NMR of compound A28

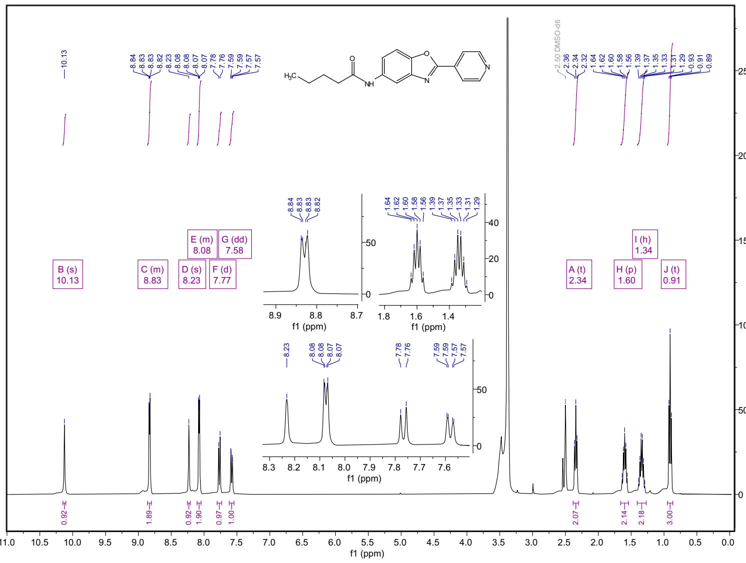

^{13}C-NMR of compound A28

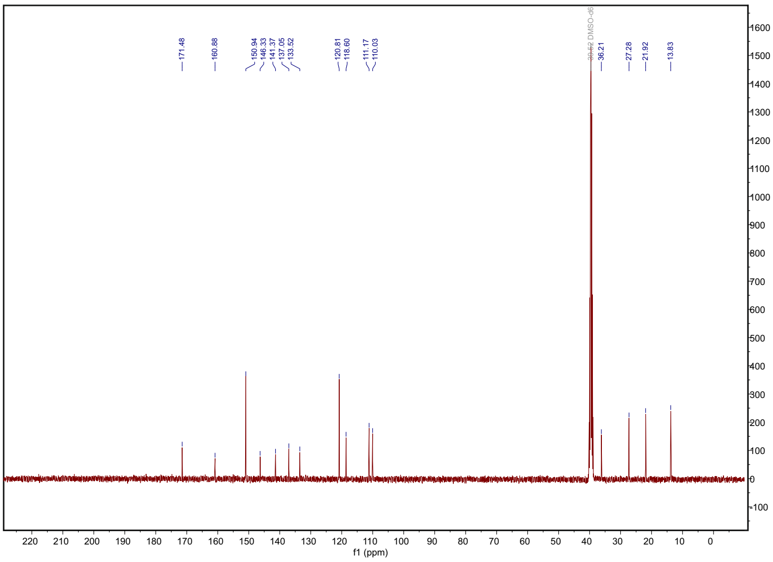

A29

^{1}H-NMR of compound A29

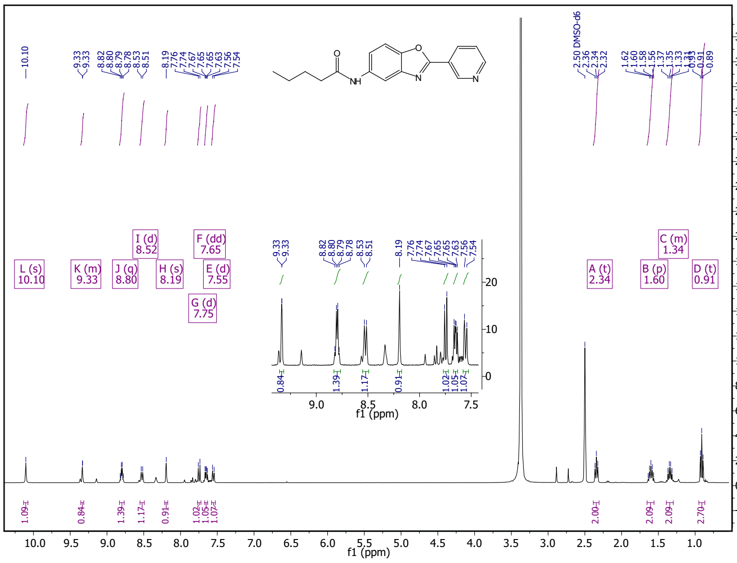

^{13}C-NMR of compound A29

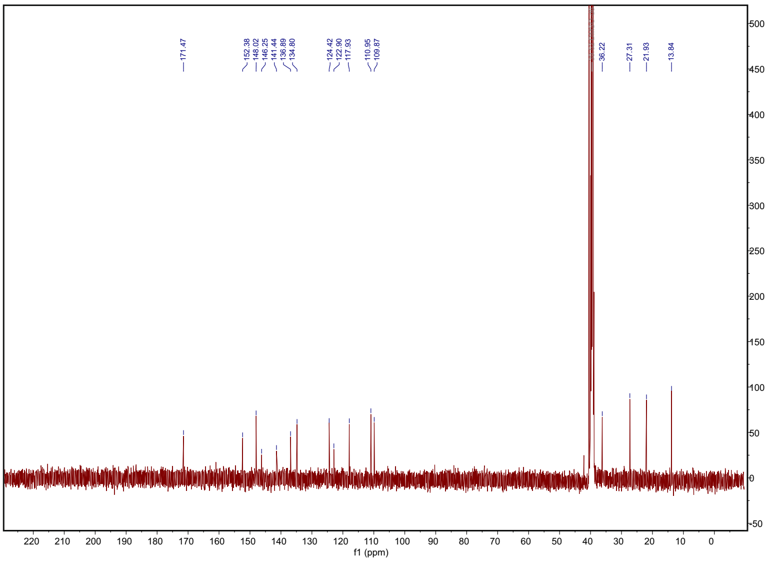

A30

^{1}H-NMR of compound A30

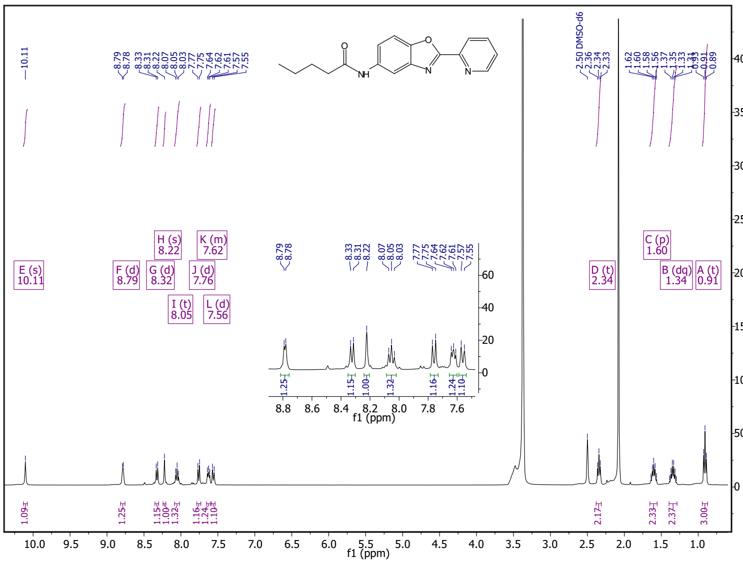

^{13}C-NMR of compound A30

No Data available

A33

^{1}H-NMR of compound A33

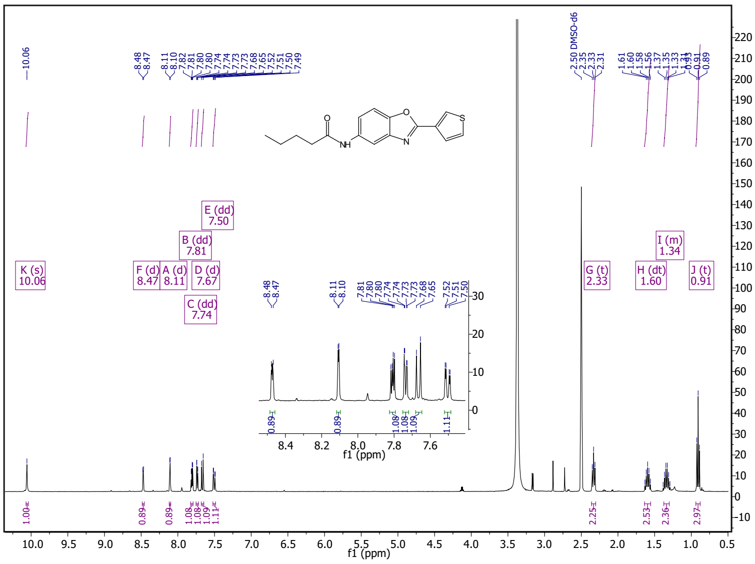

^{13}C-NMR of compound A33

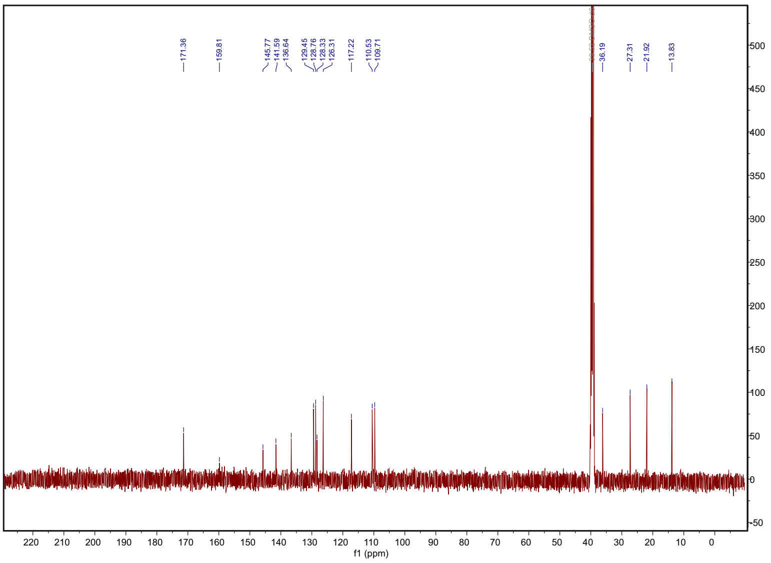

A34

^{1}H-NMR of compound A34

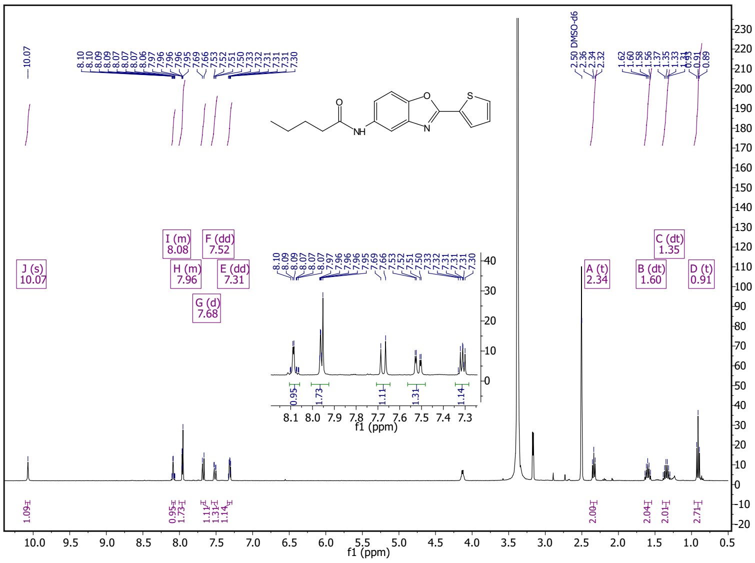

^{13}C-NMR of compound A34

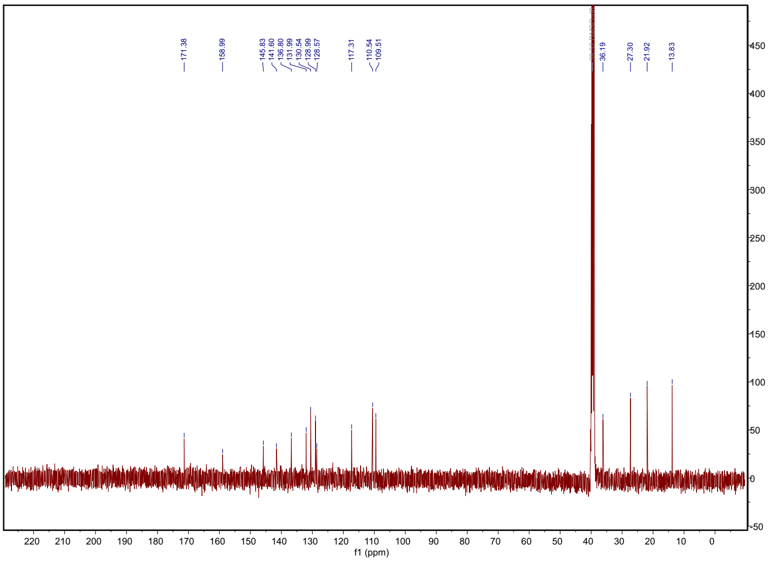

A39

^{1}H-NMR of compound A39

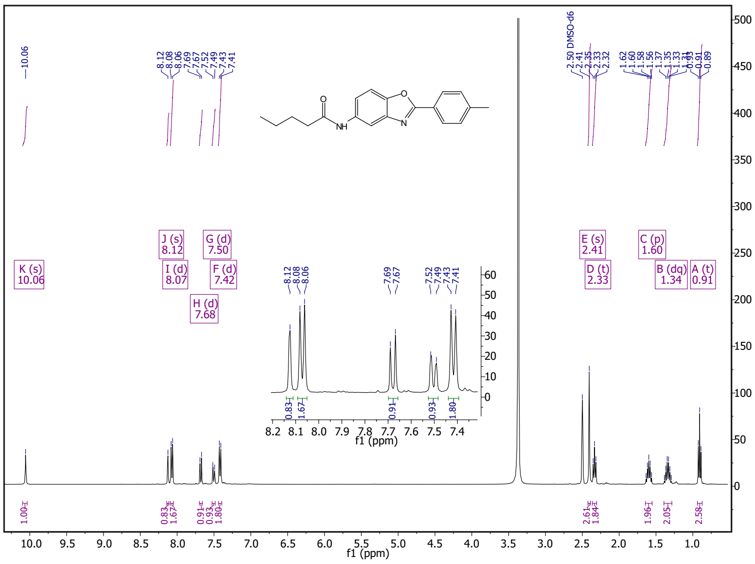

^{13}C-NMR of compound A39

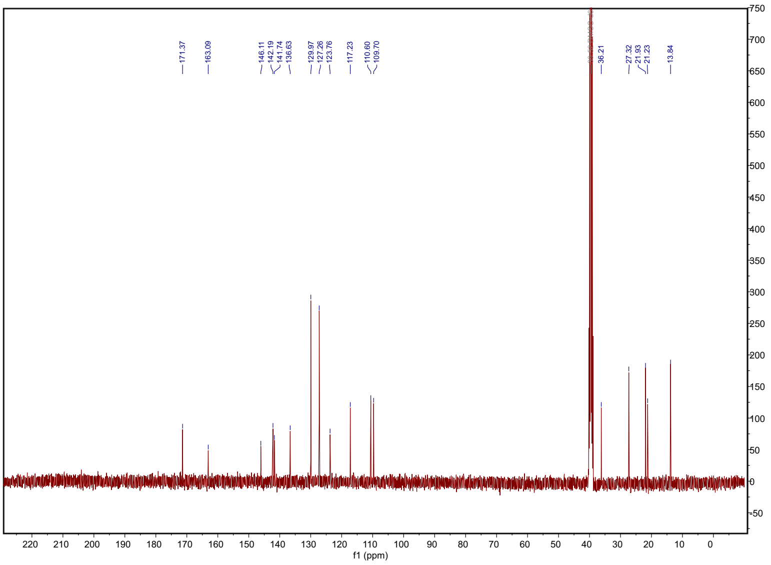

A40

^{1}H-NMR of compound A40

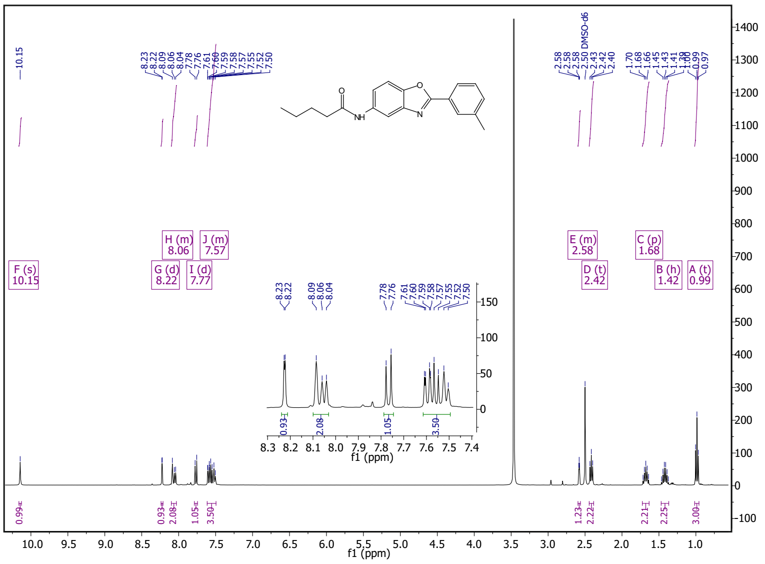

^{13}C-NMR of compound A40

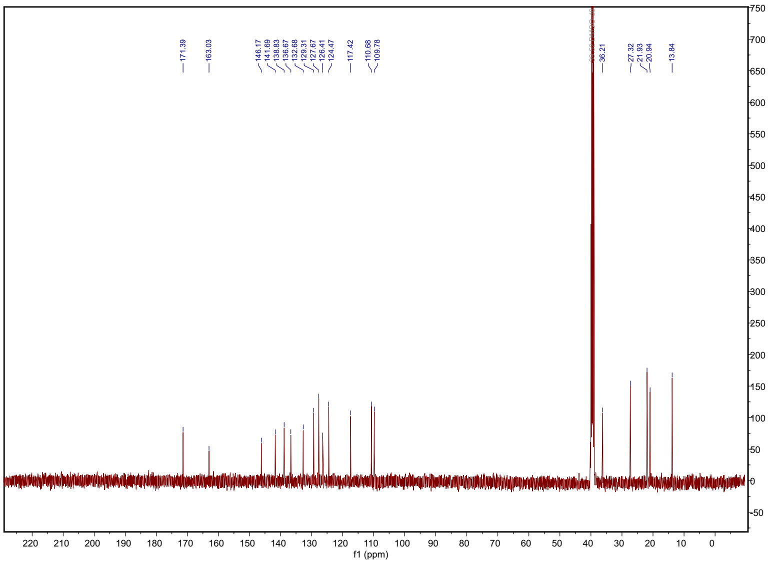

A41

^{1}H-NMR of compound A41

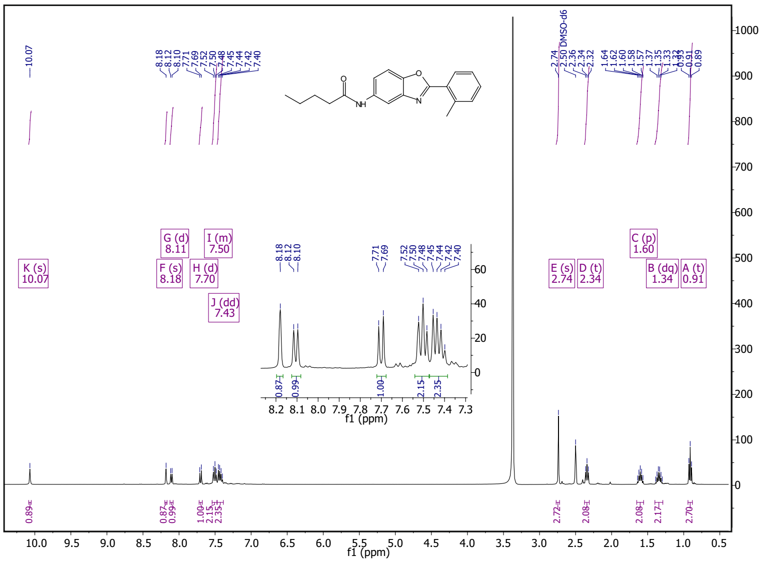

^{13}C-NMR of compound A41

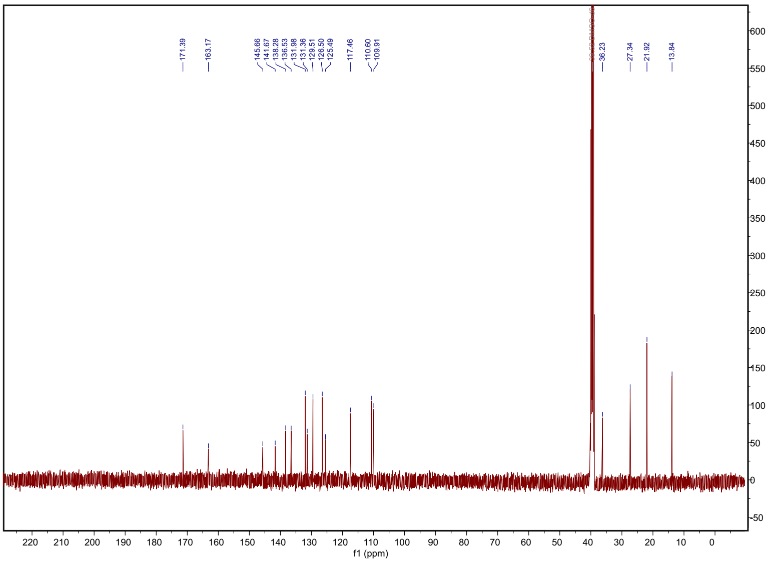

HRMS spectrum of compound A21

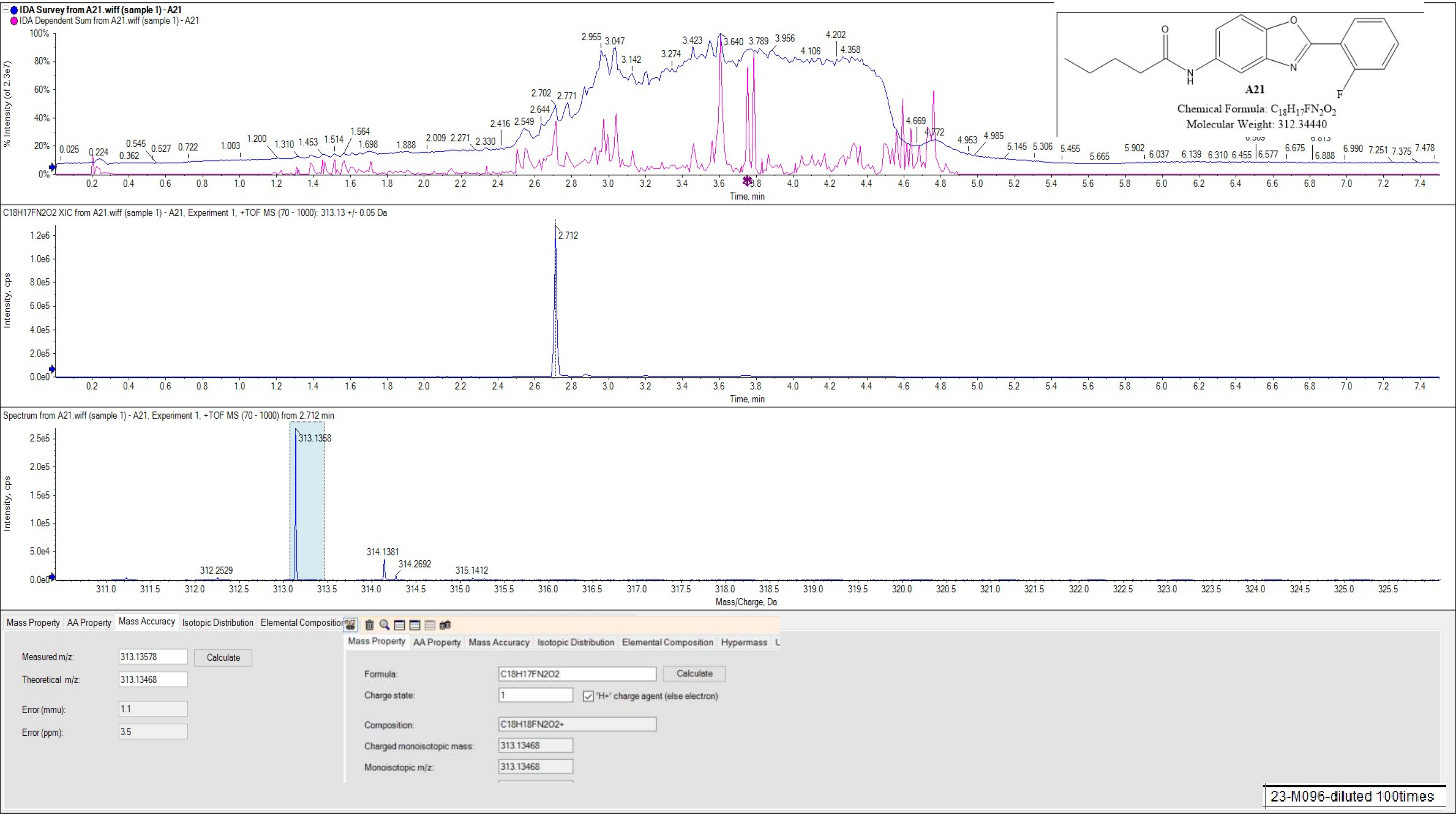

HRMS spectrum of compound A39

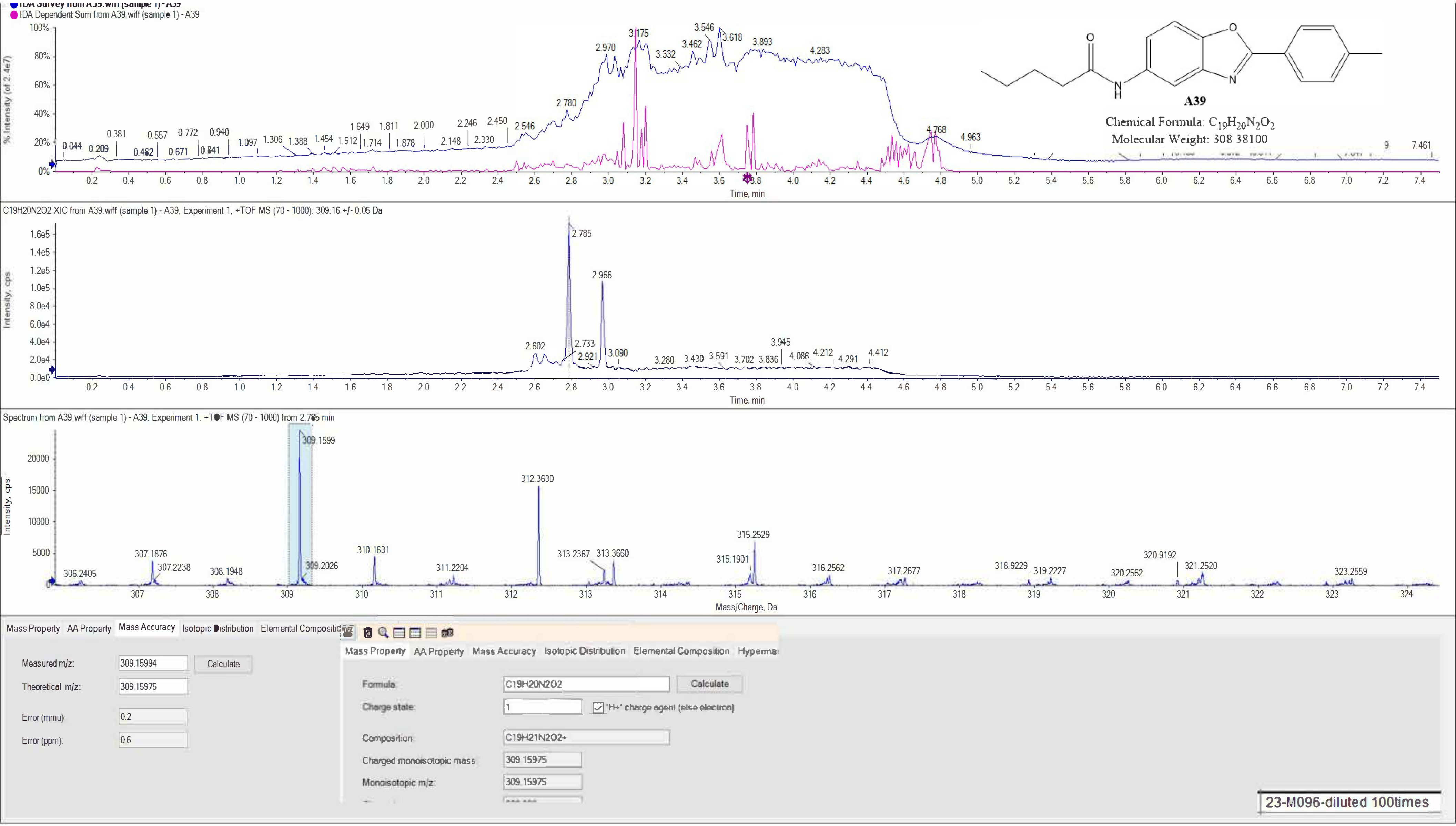

HRMS spectrum of compound A40

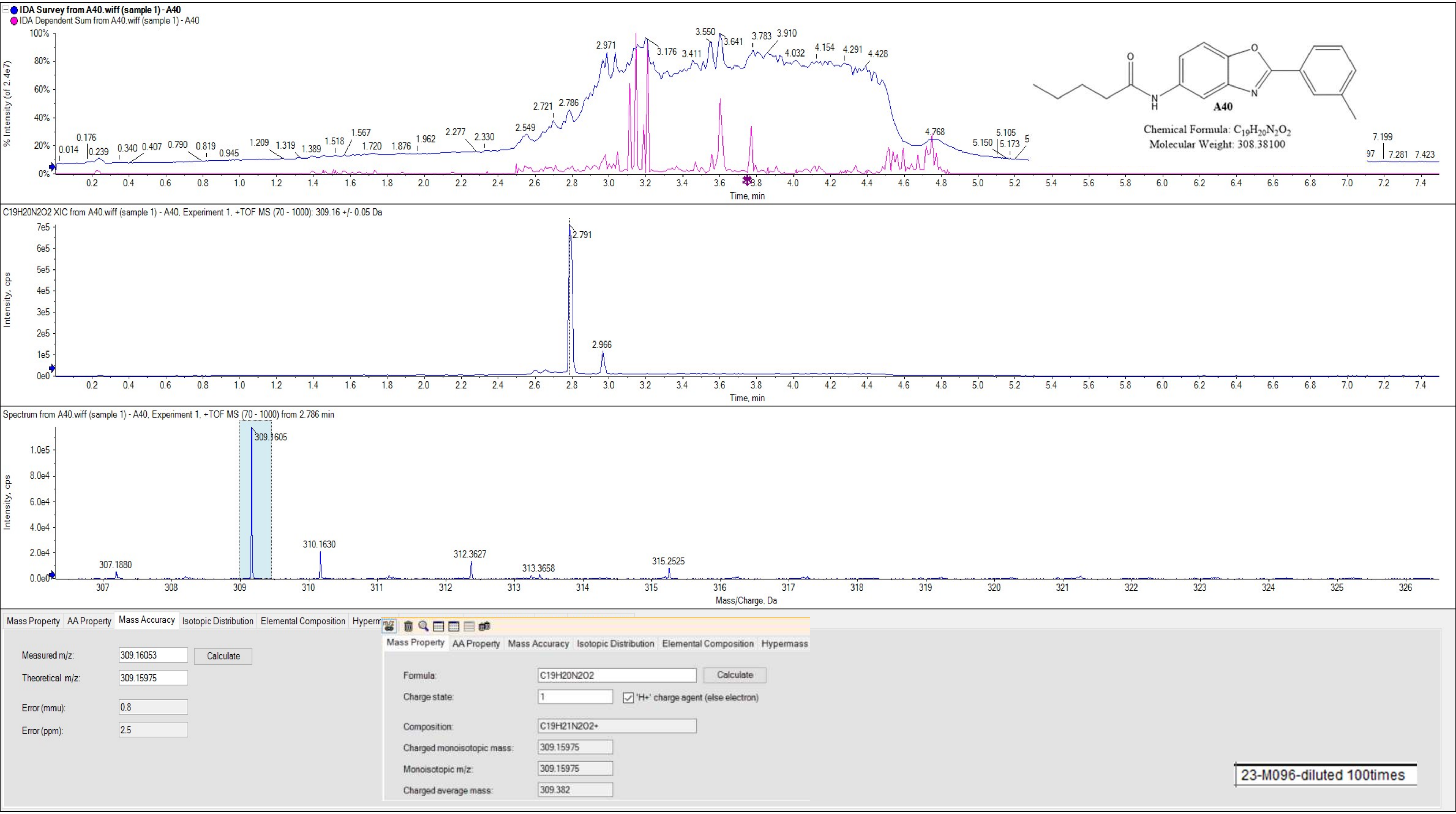

HRMS spectrum of compound A41

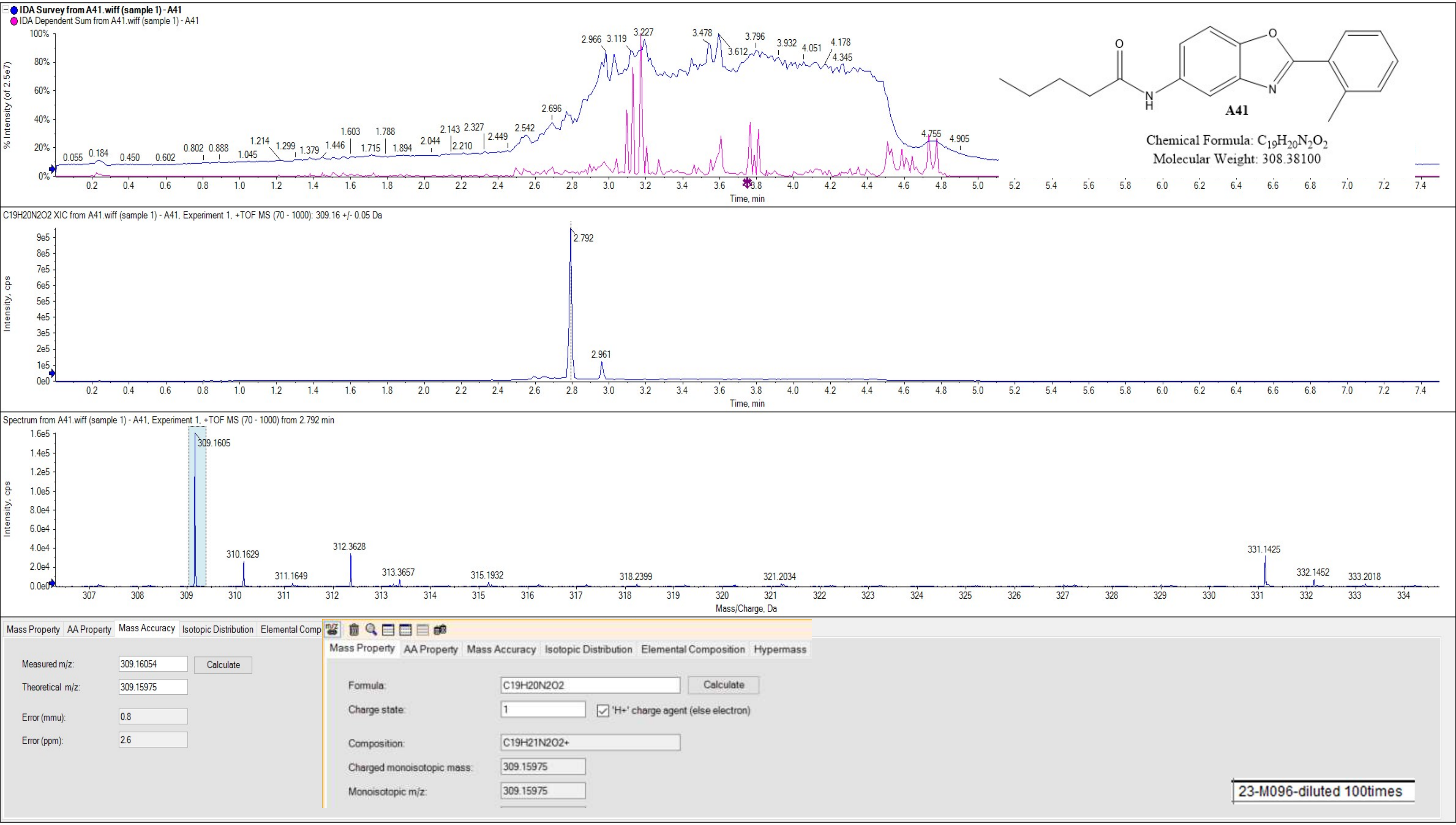

Supplement: Supplementary file 5 — Supplementary Data 2 [file 41467_2024_50076_MOESM5_ESM.pdf]
