# Supplementary material for: Benzoxazole-derivatives enhance progranulin expression and reverse the aberrant lysosomal proteome caused by GRN haploinsufficiency
Source: Nat Commun. 2024 Jul 20;15:6125. doi: 10.1038/s41467-024-50076-8 (PMC11271458; doi:10.1038/s41467-024-50076-8)
Supplement: Supplementary file 7 — Reporting Summary [file 41467_2024_50076_MOESM7_ESM.pdf]

nature portfolio

Corresponding author(s): Joachim Herz

Last updated by author(s): Jun 11, 2024

Reporting Summary

Nature Portfolio wishes to improve the reproducibility of the work that we publish. This form provides structure for consistency and transparency in reporting. For further information on Nature Portfolio policies, see our Editorial Policies and the Editorial Policy Checklist.

Statistics

For all statistical analyses, confirm that the following items are present in the figure legend, table legend, main text, or Methods section.

n/a	Confirmed	
☐	☒	The exact sample size (*n*) for each experimental group/condition, given as a discrete number and unit of measurement
☐	☒	A statement on whether measurements were taken from distinct samples or whether the same sample was measured repeatedly
☐	☒	The statistical test(s) used AND whether they are one- or two-sided<br>*Only common tests should be described solely by name; describe more complex techniques in the Methods section.*
☒	☐	A description of all covariates tested
☐	☒	A description of any assumptions or corrections, such as tests of normality and adjustment for multiple comparisons
☐	☒	A full description of the statistical parameters including central tendency (e.g. means) or other basic estimates (e.g. regression coefficient) AND variation (e.g. standard deviation) or associated estimates of uncertainty (e.g. confidence intervals)
☐	☒	For null hypothesis testing, the test statistic (e.g. *F*, *t*, *r*) with confidence intervals, effect sizes, degrees of freedom and *P* value noted<br>*Give P values as exact values whenever suitable.*
☒	☐	For Bayesian analysis, information on the choice of priors and Markov chain Monte Carlo settings
☐	☒	For hierarchical and complex designs, identification of the appropriate level for tests and full reporting of outcomes
☒	☐	Estimates of effect sizes (e.g. Cohen's *d*, Pearson's *r*), indicating how they were calculated

Our web collection on statistics for biologists contains articles on many of the points above.

Software and code

Policy information about availability of computer code

Data collection	No software was used for data collection
Data analysis	GraphPad Prism v 9.5.0 (Graphpad software), Image J (NIH), Genedata Screener Suite (genedata); SwissADME (Swiss Institute of Bioinformatics, http://www.swissadme.ch/); CorelDRAW 2020, Version 22.1.1.523 (Corell Corp.); ChemDraw Professional, Version 21.0.0.28 (PerkinElmer Informatics Inc.); ClustVis (BIIT Research Group, https://biit.cs.ut.ee/clustvis/), Proteome Discoverer v2.4 (ThermoFisher, Waltham, MA, USA), ShinyGO 0.77 (http://bioinformatics.sdstate.edu/go/)

For manuscripts utilizing custom algorithms or software that are central to the research but not yet described in published literature, software must be made available to editors and reviewers. We strongly encourage code deposition in a community repository (e.g. GitHub). See the Nature Portfolio guidelines for submitting code & software for further information.

Data

Policy information about availability of data

All manuscripts must include a data availability statement. This statement should provide the following information, where applicable:

- Accession codes, unique identifiers, or web links for publicly available datasets
- A description of any restrictions on data availability
- For clinical datasets or third party data, please ensure that the statement adheres to our policy

The data sheet for molecular structure of compounds tested by our lab, as well as where compounds were acquired and their catalog number, are included in the supplemental information as Supplementary Data 1.

The data sheet for compound NMR spectra and HRMS data is included in the supplemental information as Supplementary Data 2.

Original western blots and any additional information required to reanalyze the data reported in this paper is available on the Texas Data Repository https://doi.org/10.18738/T8/JTBZLU.

This paper does not report original code.

Research involving human participants, their data, or biological material

Policy information about studies with human participants or human data. See also policy information about sex, gender (identity/presentation), and sexual orientation and race, ethnicity and racism.

Reporting on sex and gender	*Use the terms sex (biological attribute) and gender (shaped by social and cultural circumstances) carefully in order to avoid confusing both terms. Indicate if findings apply to only one sex or gender; describe whether sex and gender were considered in study design; whether sex and/or gender was determined based on self-reporting or assigned and methods used. Provide in the source data disaggregated sex and gender data, where this information has been collected, and if consent has been obtained for sharing of individual-level data; provide overall numbers in this Reporting Summary. Please state if this information has not been collected. Report sex- and gender-based analyses where performed, justify reasons for lack of sex- and gender-based analysis.*
Reporting on race, ethnicity, or other socially relevant groupings	*Please specify the socially constructed or socially relevant categorization variable(s) used in your manuscript and explain why they were used. Please note that such variables should not be used as proxies for other socially constructed/relevant variables (for example, race or ethnicity should not be used as a proxy for socioeconomic status). Provide clear definitions of the relevant terms used, how they were provided (by the participants/respondents, the researchers, or third parties), and the method(s) used to classify people into the different categories (e.g. self-report, census or administrative data, social media data, etc.) Please provide details about how you controlled for confounding variables in your analyses.*
Population characteristics	*Describe the covariate-relevant population characteristics of the human research participants (e.g. age, genotypic information, past and current diagnosis and treatment categories). If you filled out the behavioural & social sciences study design questions and have nothing to add here, write "See above."*
Recruitment	*Describe how participants were recruited. Outline any potential self-selection bias or other biases that may be present and how these are likely to impact results.*
Ethics oversight	*Identify the organization(s) that approved the study protocol.*

Note that full information on the approval of the study protocol must also be provided in the manuscript.

Field-specific reporting

Please select the one below that is the best fit for your research. If you are not sure, read the appropriate sections before making your selection.

[x] Life sciences [ ] Behavioural & social sciences [ ] Ecological, evolutionary & environmental sciences

For a reference copy of the document with all sections, see nature.com/documents/nr-reporting-summary-flat.pdf

Life sciences study design

All studies must disclose on these points even when the disclosure is negative.

Sample size	In vitro samples sizes were based on sample sizes used in our previous work to determine regulators of PGRN (Cenik et al., 2011). In vivo sample sizes were based on availability of mice with age matched controls. All sample sizes for each experiment are indicated in the method section.
Data exclusions	No data was excluded
Replication	All data sets shown include all replicates tested.
Randomization	Mice were selected at random based on age and genotype
Blinding	No imaging or behavioral data was assessed, therefore blinding was not performed.

Reporting for specific materials, systems and methods

We require information from authors about some types of materials, experimental systems and methods used in many studies. Here, indicate whether each material, system or method listed is relevant to your study. If you are not sure if a list item applies to your research, read the appropriate section before selecting a response.

Materials & experimental systems

n/a	Involved in the study	
	x	Antibodies
	x	Eukaryotic cell lines
x		Palaeontology and archaeology
	x	Animals and other organisms
x		Clinical data
x		Dual use research of concern
x		Plants

Methods

n/a	Involved in the study	
x		ChIP-seq
x		Flow cytometry
x		MRI-based neuroimaging

Antibodies

Antibodies used	Anti-GRN 1:1,500 dilution, (sigma HPA008763, Llot 000043984); Anti-granulin 1:3,000 (Abcam ab252834, Llot GR334867-1); Anti-beta actin 1:5,000-1:20,000 dilution, (Abcam ab8227, GR342990-1); Anti-HA 1:1000 dilution (Cell Signaling 3724S, Lot 10); Anti-mouse CD107a 1:1000 dilution (BD Biosciences 553792, Lot 0000055197); Anti-EEA1 1:1000 dilution (Cell Signaling 3288S, Lot 8); Anti-Rab7 1:1000 dilution (Cell Signaling 9367S, Lot 3); Anti-GM130 1:1000 dilution (Thermo Fisher MA5-35107, Lot XD3555932); Anti-COX IV 1:1000 dilution (Cell Signaling 4850S, Lot 10); Anti-Lamin A+C 1:1000 dilution (Abcam ab133256, Lot 7); Anti-Calnexin 1:12,000 dilution (Cell Signaling 2679S, Lot 03011836); Anti-TPP1 1:100 dilution (Santa Cruz sc-393961, Lot L1621); Secondary antibodies: HRP Anti-rabbit IgG 1:2000-1:20,000 dilution (Cytivia NA9341ML, Llot 18111932); HRP anti-rat IgG 1:5,000 dilution (Biolegend 405405, Llot B364032); IRDye 800CW anti-rabbit IgG 1:20,000 dilution (Li-cor 926-32213, Lot D30627-15); IRDye 800CW donkey anti-mouse IgG 1:20,000 dilution (Li-Cor 926-32212, Lot C61116-02); IRDye 680RD donkey anti-rabbit IgG 1:20,000 dilution (Li-Cor 926-68073, Llot D30627-15); IRDye 680RD goat anti-rat IgG 1:20,000 dilution (Li-Cor 926-68076, Lot AB_10956590).
Validation	All antibodies were validated by the indicated companies, but granulin/progranulin antibodies were additionally validated with our own KO samples before experimental use.

Eukaryotic cell lines

Policy information about cell lines and Sex and Gender in Research

Cell line source(s)	Mouse: Neuro2A ATCC CCL-131; Human: U-373 ATCC HTB-17; Human: HDF 2 UCSF MAC; HDF X UCSF MAC FTD25; HDF Y UCSF MAC FTD23
Authentication	Cell lines were not authenticated
Mycoplasma contamination	Cell lines were not tested for mycoplasma contamination
Commonly misidentified lines (See ICLAC register)	No commonly misidentified cell lines are used

Animals and other research organisms

Policy information about studies involving animals; ARRIVE guidelines recommended for reporting animal research, and Sex and Gender in Research

Laboratory animals	in general GRN mutant and wild type mice are on mixed 129SvEv Bradley;C57BL/6J background, for ICV pump Implantation experiment occurred at 3 months (±5 days) in 21 male, Grn+/+ or Grn+/- mice. IP injections were performed on 66 three-month old, male Grn wild-type and mutant mice. Pharmacokinetic analysis of IP injections were performed with 21 female CD1 mice, Pharmacokinetic analysis of OP dosage were performed with 21 female CD1 mice
Wild animals	*Provide details on animals observed in or captured in the field; report species and age where possible. Describe how animals were caught and transported and what happened to captive animals after the study (if killed, explain why and describe method; if released, say where and when) OR state that the study did not involve wild animals.*
Reporting on sex	All experimental animals were male, except for the parmacokinetic studies, here female mice were used
Field-collected samples	*For laboratory work with field-collected samples, describe all relevant parameters such as housing, maintenance, temperature, photoperiod and end-of-experiment protocol OR state that the study did not involve samples collected from the field.*
Ethics oversight	All procedures were performed in accordance with the protocols approved by the Institutional Animal Care and Use Committee of the University of Texas Southwestern Medical Center. Protocol: IACUC APN 2015-101088-G

Note that full information on the approval of the study protocol must also be provided in the manuscript.

Plants

Seed stocks	*Report on the source of all seed stocks or other plant material used. If applicable, state the seed stock centre and catalogue number. If plant specimens were collected from the field, describe the collection location, date and sampling procedures.*
Novel plant genotypes	*Describe the methods by which all novel plant genotypes were produced. This includes those generated by transgenic approaches, gene editing, chemical/radiation-based mutagenesis and hybridization. For transgenic lines, describe the transformation method, the number of independent lines analyzed and the generation upon which experiments were performed. For gene-edited lines, describe the editor used, the endogenous sequence targeted for editing, the targeting guide RNA sequence (if applicable) and how the editor was applied.*
Authentication	*Describe any authentication procedures for each seed stock used or novel genotype generated. Describe any experiments used to assess the effect of a mutation and, where applicable, how potential secondary effects (e.g. second site T-DNA insertions, mosiacism, off-target gene editing) were examined.*
